# Targeting Vascular Endothelial Growth Factor Receptor 2 (VEGFR-2): Latest Insights on Synthetic Strategies

**DOI:** 10.3390/molecules29225341

**Published:** 2024-11-13

**Authors:** Carolina S. Marques, Pedro Brandão, Anthony J. Burke

**Affiliations:** 1 LAQV-REQUIMTE, Institute for Research and Advanced Training, University of Évora, Rua Romão Ramalho, 59, 7000-641 Evora, Portugal; 2 Egas Moniz Center for Interdisciplinary Research (CiiEM), Egas Moniz School of Health & Science, Campus Universitátio, Quinta da Granja, Monte da Caparica, 2829-511 Caparica, Portugal; 3 Centro de Química de Coimbra, Institute of Molecular Sciences (CQC-IMS), Departamento de Química, Faculdade de Ciências e Tecnologia, University of Coimbra, 3004-535 Coimbra, Portugal; 4 iBB-Institute for Bioengineering and Biosciences, Department of Bioengineering, Associate Laboratory i4HB–Institute for Health and Bio-Economy, Instituto Superior Técnico, University of Lisboa, Av. Rovisco Pais, 1049-001 Lisbon, Portugal; 5 Faculty of Pharmacy, University of Coimbra, Pólo das Ciências da Saúde, 3000-548 Coimbra, Portugal

**Keywords:** VEGFR-2, inhibitors, 5-member heterocycle, 6-member heterocycle, coumarin, isatin, urea, thiourea

## Abstract

Vascular endothelial growth factor receptor 2 (VEGFR-2) is a crucial mediator of angiogenesis, playing a pivotal role in both normal physiological processes and cancer progression. Tumors harness VEGFR-2 signaling to promote abnormal blood vessel growth, which is a key step in the metastasis process, making it a valuable target for anticancer drug development. While there are VEGFR-2 inhibitors approved for therapeutic use, they face challenges like drug resistance, off-target effects, and adverse side effects, limiting their effectiveness. The quest for new drug candidates with VEGFR-2 inhibitory activity often starts with the selection of key structural motifs present in molecules currently used in clinical practice, expanding the chemical space by generating novel derivatives bearing one or more of these moieties. This review provides an overview of recent advances in the development of novel VEGFR-2 inhibitors, focusing on the synthesis of new drug candidates with promising antiproliferative and VEGFR-2 inhibition activities, organizing them by relevant structural features.

## 1. Introduction

Vascular endothelial growth factor receptor 2 (VEGFR-2) plays a central role in angiogenesis, the process of new blood vessel formation. This process, under physiological conditions, is crucial for processes such as wound healing and tissue regeneration. However, in diseases such as cancer, VEGFR-2 becomes a major player in supporting tumor growth and metastasis by promoting an abnormal and excessive blood supply [1,2,3]. VEGFR-2, as a key mediator of angiogenesis, has emerged as a relevant target for medicinal chemists aiming to develop new anticancer therapies. VEGFR-2 inhibitors can disrupt the tumor’s blood supply and inhibit its progression, as blocking VEGFR-2 has the potential to halt angiogenesis, making it a valuable strategy in the fight against cancer. These inhibitors are often designed via molecular hybridization to include antiproliferative activity, enhancing their anticancer potential [4,5].

The expression of the VEGF gene is induced by hypoxia and affected by other growth factors. This upregulation leads to a cascade of events, which can include vascular permeability increase, endothelial cell sprouting, and the expression of tissue matrix metalloproteinases. The proangiogenic effect is observed through vessel formation and the expansion of the vascular network. Small-molecule VEGFR-2 inhibitors often target the ATP binding site of the receptor, competing with the ATP molecule. Since the catalytic domain of VEGFR-2 is similar to that of several other receptors that use ATP as a substrate, VEGFR-2 inhibitors are often multitarget inhibitors, inhibiting other VEGFR and other receptors that are present in several tissues, including healthy ones. The therapeutic advantage of these inhibitors relies on the overexpression of VEGFR-2 in the cancer cells of multiple cancers [6,7].

According to the mechanism of action, VEGFR-2 inhibitors can be subdivided into three types: Type I inhibitors can establish one to three hydrogen bonds with the receptor’s active site; Type II inhibitors are allosteric inhibitors that bind to a hydrophobic pocket adjacent to the adenosine binding site; and Type III are covalent. [8] New potential drug candidates are often developed using chemical moieties in known inhibitors, aiming to establish more efficient drug–target interactions, higher target selectivity (to reduce potential side effects), and better pharmacokinetic profiles, circumventing drug-resistance mechanisms. 

**Figure 1:**
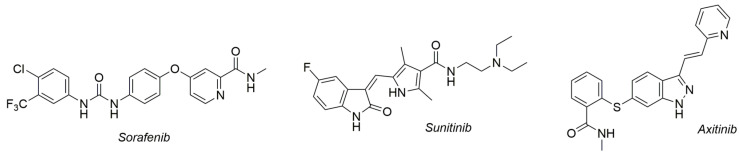
Examples of VEGFR-2 inhibitor drugs used in clinical practice.

Several VEGFR-2 inhibitors have already been introduced into clinical practice, offering promising treatment options for various types of cancer. Tyrosine kinase inhibitors (TKIs) such as sorafenib, sunitinib, and axitinib (Figure 1) target the VEGFR-2 signaling pathway and are commonly used in the treatment of cancers such as renal cell carcinoma, hepatocellular carcinoma, and gastrointestinal stromal tumors [9]. These inhibitors have demonstrated efficacy in slowing disease progression and, in some cases, extending patient survival. However, despite their clinical success, VEGFR-2 inhibitors also face limitations, such as the development of resistance, off-target effects, and adverse reactions like hypertension and cardiotoxicity, which hinder their long-term use [7,10]. The development of drug resistance can be multifactorial and occur due to the existence of redundant signaling pathways within the cells, the selection of malignant clones that exhibit hypoxia resistance, and the increase in the level of circulating nontumor proangiogenic factors [6].

Given the challenges associated with the current VEGFR-2 inhibitors, there is a growing need to discover and develop novel agents with improved specificity, reduced side effects, and the ability to overcome resistance mechanisms [11,12]. For synthetic organic chemists and medicinal chemists, the continuous search for new synthetic strategies that target VEGFR-2 is essential to address the unmet clinical needs in cancer therapy. Advances in rational drug design, structure-based optimization, and molecular hybridization have the potential to yield next-generation inhibitors that can offer enhanced efficacy, safety, and therapeutic outcomes. In this work, we perform a detailed review of the latest reports on VEGFR-2 inhibitor synthesis, dividing them according to relevant structural features. The synthetic strategies and bioactivity evaluations are showcased, highlighting the recent efforts in this growing field in synthetic organic chemistry, medicinal chemistry, and pharmacology.

## 2. Heterocyclic Key Scaffolds as Promising VEGFR-2 Inhibitors

In the following section, we will present the most recent discoveries from the last 10 years regarding potential small molecules as VEGFR-2 inhibitors. We will discuss their synthetic pathways, biological behaviors, and other topics of interest. Considering the molecular structures of the numerous VEGFR-2 inhibitors that are commercially available and others in the development pipeline, this section will be organized according to the main heterocyclic scaffold of these potential small molecules.

### 2.1. Five-Membered Ring Heterocycles

#### 2.1.1. Five-Membered Ring Heterocycles with One, Two, and Three Heteroatoms

Moieties such as the pyrrole ring are present in clinically approved VEGFR-2 inhibitors, namely sunitinib.

**Scheme 1:**
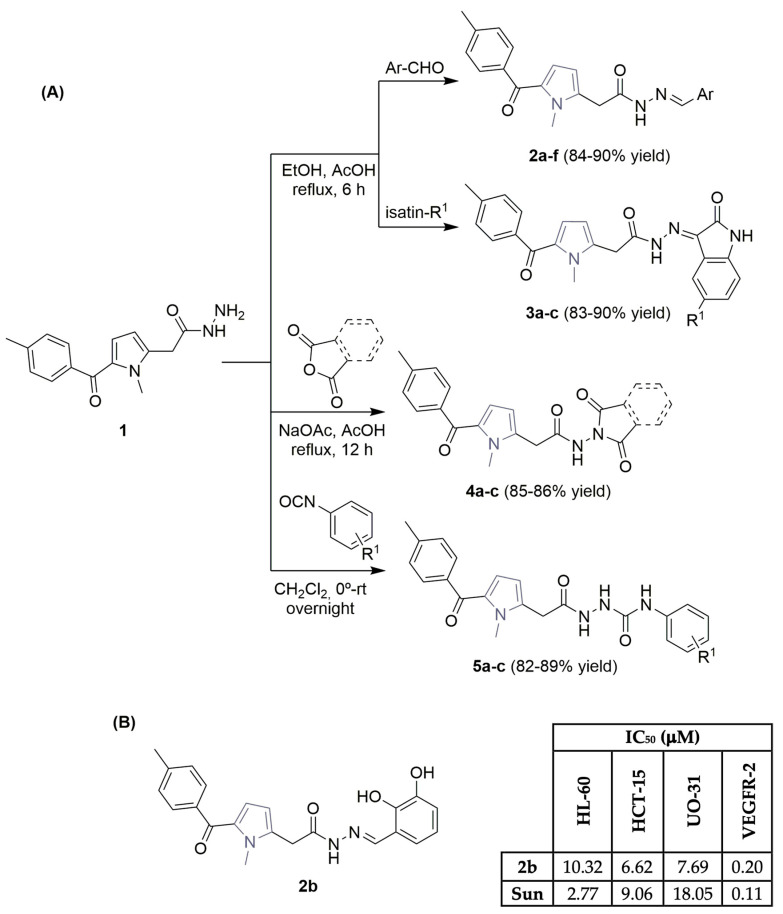
Synthesis (**A**) and biological profile (**B**) of tolmetin derivatives possessing a pyrrole ring and an acetamide moiety in their scaffold. (HL-60: leukemia cell line; HCT-15: colon cancer cells; UO-31: renal cancer cells; Sun: sunitinib).

Recently, Kassab et al. [13] documented the synthesis of new tolmetin derivatives, based on the key pharmacophoric features of sunitinib (Figure 1) with a pyrrole ring and an acetamide moiety, targeting the development of efficient anticancer agents, by inhibiting VEGFR-2. Using previously described literature procedures [14], the synthesis of fifteen new tolmetin derivatives was carried out using tolmetin hydrazide **1**, achieving good yields and non-chromatographic reaction conditions (Scheme 1A). ^1^H NMR analysis of the tolmetin hydrazone derivatives **2a**–**f** and **6a**–**c** indicated the presence of tautomers (keto amide and enol amide mixtures). An external company screened all the synthesized compounds on a panel of 60 tumor cell lines, representing lung, colon, CNS, melanoma, ovarian, renal, prostate, breast, and leukemia cancers. The preliminary growth inhibition percentage (GI%) achieved by the tested compounds identified compounds **2b** and **2c** as the most active. In particular, compound **2b**, a tolmetin derivative featuring an azomethine linker directly connected to a 2,3-dihydroxyphenyl group, was the most potent against three human tumor cell lines, namely, leukemia (HL-60), colon (HCT-15), and renal (UO-31) cell lines (Scheme 1B), with IC_50_ values of 10.32, 6.62 and 7.69 μM, respectively, closely matching those of *sunitinib* (Figure 1), used as the positive control. An IC_50_ value of 0.20 μM was obtained for compound **2b** in the *in vitro* cell-based VEGFR-2 kinase inhibitory assay (Scheme 1B), with molecular docking calculations suggesting promising binding configurations of the scaffold of **2b** with key amino acids in the VEGFR-2 binding site. Other biological assays of interest, like the Annexin V-FITC apoptosis assay, revealed that the antiproliferative behavior of **2b** in cell death is due to physiological apoptosis.

**Scheme 2:**
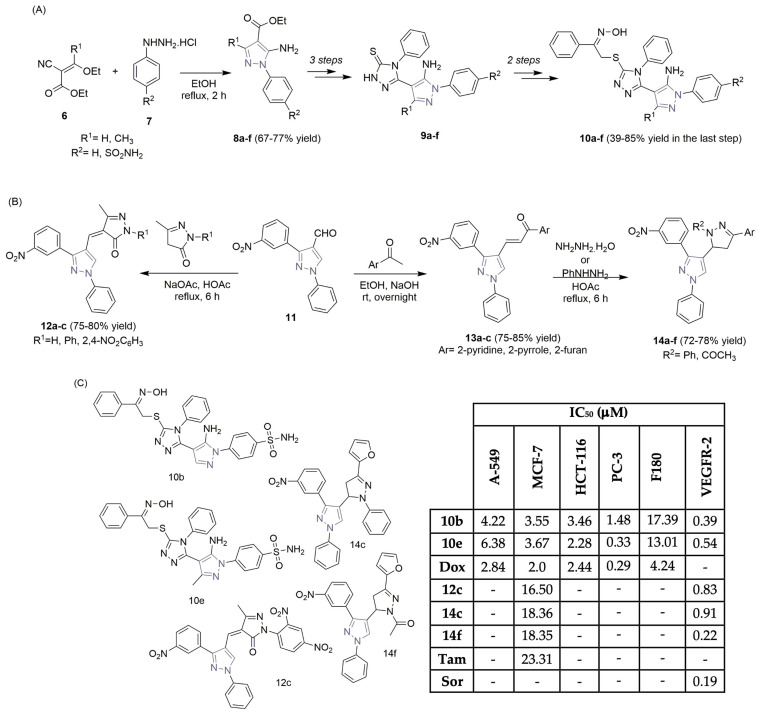
Synthesis of pyrazole-hybrid scaffolds (**A**,**B**) and the biological profiles (**C**) of the most potent ones. (A-549: lung cancer cells; MCF-7: breast cancer cells; HCT-116: colon cancer cells; PC-3: prostate cancer cells; F180: normal fibroblasts; Dox: doxorubicin; Tam: tamoxifen; Sor: sorafenib).

Abdellatif et al., in research aimed at synthesizing new non-steroidal anti-inflammatory drugs and their potential to inhibit cyclooxygenase enzymes, described the synthesis and bio-evaluation of new pyrazole hybrids (Scheme 2A,C) [15]. Using the literature findings, the pyrazole esters **8a**–**f** were obtained in moderate yields through the cyclization reaction of ester **6**, with the appropriate phenylhydrazine **7**. Hydrazinolysis, cyclization, and other key reaction steps lead to the formation of the new pyrazole-1,2,4-triazole hybrids **10a**–**f**, achieving good reaction outcomes. Antiproliferative assays were performed on several tumor cell lines (lung, breast, colon, and prostate) using some of the new scaffolds, revealing high potency for two sulfamoyl thioethanone oxime derivatives, **10b** and **10e**, particularly the prostate cancer cell line PC-3 (with IC_50_ values of 1.48 and 0.33 μM, respectively; Scheme 2C). *Doxorubicin* (a chemotherapeutic agent) was used as a reference control. The behaviors of pyrazole hybrids **10b** and **10e** were assessed against F180 fibroblasts to check selectivity indicators. Compared to PC-3 cells, used as controls (VEGFR-2 (Fold): 1), the good inhibitory activities of **10b** and **10e** against VEGFR-2 (Fold) were confirmed (0.39 and 0.54, respectively; see Scheme 2C). Docking studies were carried out on the epidermal growth factor receptor (EGFR) enzyme, involved in tumor initiation, angiogenesis, and metastasis, demonstrating that the internal oxime moiety of the scaffolds **10b** and **10e** forms extra hydrogen bonds with specific amino acids in the receptor’s cleft.

Dawood and co-workers—considering the value of the pyrazole ring in VEGFR-2 inhibition and cancer control—described the synthesis of new pyrazole hybrids, coupled with pyrazoline, thiazolopyrimidine, and pyrazolone moieties, and evaluated their biological profiles against breast cancer cells (MCF-7) (Scheme 2B,C) [16]. Using 3-(3-nitrophenyl)-1-phenyl-1H-pyrazole-4-carbaldehyde **11**, it was easy to obtain the pyrazole–pyrazolone hybrids **12a**–**c** through a Knoevenagel condensation reaction with pyrazolone derivatives, achieving good reaction yields (Scheme 2B). *N*-phenyl and *N*-acetyl-pyrazole-pyrazoline hybrids **14a**–**f** were easily obtained from precursor **11** via Claisen condensation, yielding the chalcone derivatives **13a**–**c**, followed by the Michael addition reaction of hydrazine hydrate and substituted hydrazines, and the subsequent dehydration and cyclization steps (Scheme 2B). The pyrazoline derivatives **14c** and **14f** and the pyrazolone derivative **12c** revealed significant activity toward MCF-7 cells, with IC_50_ values of 18.36, 18.35, and 16.50 μM, respectively. Tamoxifen was used as the reference drug, with an IC_50_ value of 23.31 μM (Scheme 2C). Additionally, the most potent analogs were tested for their VEGFR-2 inhibitory activity, demonstrating a significant reduction of VEGFR-2 in MCF-7 cells (72–79%), compared to the control cells. Using sorafenib (Figure 1) as the control drug (IC_50_ of 0.19 μM), compounds **12c**, **14c**, and **14f** revealed high inhibitory competence against VEGFR-2 (IC_50_ values of 0.83, 0.91, and 0.22 μM, respectively; see Scheme 2C). Molecular docking simulations showed that compounds **12c**, **14c**, and **14f** bind properly to the active site of the VEGFR-2 enzyme.

**Scheme 3:**
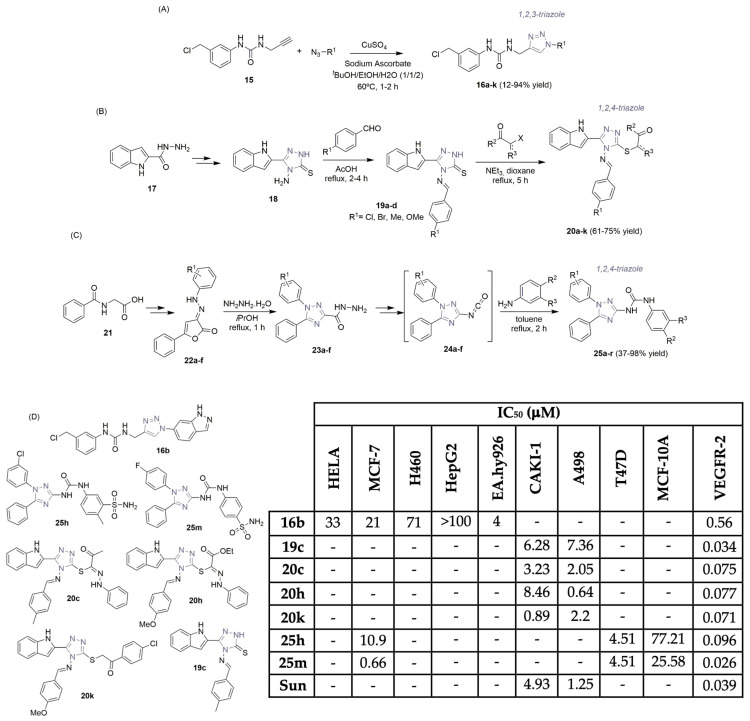
Synthesis of triazole-hybrid scaffolds (**A**–**C**) and biological profiles (**D**) of the most potent derivatives. (HELA: cervical cancer cells; MCF-7 and T47D: breast cancer cells; H460: lung cancer cells; HepG2: hepatic carcinoma cells; EA.hy926: vascular endothelial cells; CAKI-1 and A498: renal cancer cells; MCF-10A: non-tumorigenic breast normal cells; Sun: sunitinib).

Triazole units, due to their remarkable biological profile and easy synthetic access, have also received appreciable attention in drug discovery research for cancer therapy [17,18]. In the last ten years, three research groups described their findings concerning VEGFR-2 inhibition with new 1,2,3- and 1,2,4-triazole hybrids (Scheme 3). Vajragupta and co-workers described the synthesis of a small library of 1,4-(disubstituted)-1*H*-1,2,3-triazoles, **16a**–**k** (Scheme 3A), taking into account previous research data regarding EGFR inhibition [19]. After effective in silico experiments to identify the best scaffold, eleven virtual hits were selected, synthesized, and screened for kinase inhibition. The well-known Cu(I)-catalyzed azide-alkyne cycloaddition reaction (click-chemistry) was used efficiently to obtain the 1,2,3-triazole-hybrids in low to excellent yields, using a 3-(3′-chloromethylphenyl) urea **15** building block (Scheme 3A). Only one compound, the 6-indazolyl triazole derivative **16b**, showed inhibition of VEGFR-2, at 1 μM. The IC_50_ value of **16b** against VEGFR-2 was 0.56 μM (Scheme 3D). Antiproliferative activity of **16b** was also screened in four cancer cell lines: cervical, breast, lung, and hepatic carcinoma (HELA, MCF-7, H460, and HepG2, respectively; see Scheme 3D). Also, **16b** was screened against human umbilical vein endothelial cells (Ea.hy926) for antiangiogenic effects, exhibiting high selectivity (IC_50_ of 4 μM; see Scheme 3D). Docking studies indicated the importance of the 6-indazolyl moiety of **16b**, identifying two hydrogen bond interactions with key amino acid residues in the front pocket of the VEGFR-2 enzyme.

Farghaly and co-workers, within the scope of their research work, considering drug discovery in renal cancer, described the synthesis and biopotential of a library of new indolyl-1,2-4-triazole derivatives **20a**–**k** (Scheme 3B,D) [20]. Starting from 1*H*-indole-2-carbohydrazide derivative **17**, the aminothione precursor **18** was obtained successfully in two reaction steps. A condensation reaction using a set of aromatic aldehydes yielded the 5-(1*H*-indol-2-yl)-4-((4-substitutedbenzylidene)amino)-2,4-dihydro-3*H*-1,2,4-triazole-3-thione derivatives **19a**–**d** in moderate yields (68–75%). Derivatives **19a**–**d** underwent a nucleophilic addition reaction with hydrazonoyl chlorides and phenacyl bromines, yielding the target indolyl-1,2,4-triazole hybrids **20a**–**k** in moderate yields (Scheme 3B). The library was screened *in vitro* for their VEGFR-2 inhibitory activity using sunitinib (Figure 1) as a reference drug, and compounds **19c**, **20c**, **20h**, and **20k** presented effective anti-VEGFR-2 activity (IC_50_ values of 0.034–0.075 μM; see Scheme 3D). These compounds were screened against two human renal cancer cell lines (CAKI-1 and A498) with IC_50_ values ranging from sub-micromolar to low micromolar levels (IC_50_ values of 0.64–8.46 μM; see Scheme 3D). Compound **20k**, bearing a 4-chlorophenacyl moiety at the S atom of the triazolethione core, was five times more potent than the control, sunitinib (Figure 1), against the CAKI-1 cell line (0.89 μM compared to 4.93 μM, respectively; see Scheme 3D). The same behavior was noticed for compound **20h** compared to the A498 cell line (with an IC_50_ of 0.64 μM for 20h compared to an IC_50_ of 1.25 μM for sunitinib; see Scheme 3D). Cytotoxicity tests were performed on RPTEC/TERT1 non-cancer human renal cells using compounds **20c** and **20k**, revealing IC_50_ values of 52.5 and 25.4 μM, demonstrating a better safety profile than the reference drug sunitinib (Figure 1) (IC_50_ of 15.3 μM). Docking studies revealed strong hydrogen bonding and hydrophobic interactions with the key residues within the active site of VEGFR-2.

Recently, Supuran, Eldehna, et al. documented their latest discoveries regarding new VEGFR-2 inhibitors [21]. The synthesis of a library of 1,5-diaryl-1,2,4-triazole ureas, labeled **25a**–**r**, was accomplished, starting from hippuric acid **21**. Cyclization and coupling reactions with diazonium salts led to the hydrazone intermediates **22a**–**f**, followed by the corresponding formation of the hydrazides **23a**–**f** with hydrazine monohydrate through the Sawdey rearrangement. Two-step reactions, including a Curtius rearrangement, resulted in the formation of the 3-isocyanato-1,5-diphenyl-1*H*-1,2,4-triazole derivatives **24a**–**f**. In order to obtain the corresponding urea-linker tethered products **25a**–**r**, benzenesulfonamides were used to react with the former intermediates **24a**–**f** (Scheme 3C). After the preliminary inhibitory evaluation was performed against 60 cancer cell lines, a few of these compounds were assessed for *in vitro* VEGFR-2 inhibitory potential. Compounds **25h** and **25m** were the most potent, with IC_50_ values of 0.096 and 0.026 μM (Scheme 3D). Compound **25m**, with a 4-fluorophenyl unit, revealed better VEGFR-2 inhibitory activity than sunitinib (Figure 1), the reference drug (IC_50_ of 0.039 μM; see Scheme 3D). Those compounds were screened against breast cancer cell lines (MCF-7 and T47D), exhibiting promising activity (with a remarkable IC_50_ value of 0.66 μM for 25m in MCF-7 cells; see Scheme 3D). Nontumorigenic MCF-10A cells were also tested and supported the noteworthy selectivity of compound **25m**, with a selectivity index of 38.76. Docking studies investigated the binding modes of **25m**, supporting the obtained VEGFR-2 inhibitory activity.

**Scheme 4:**
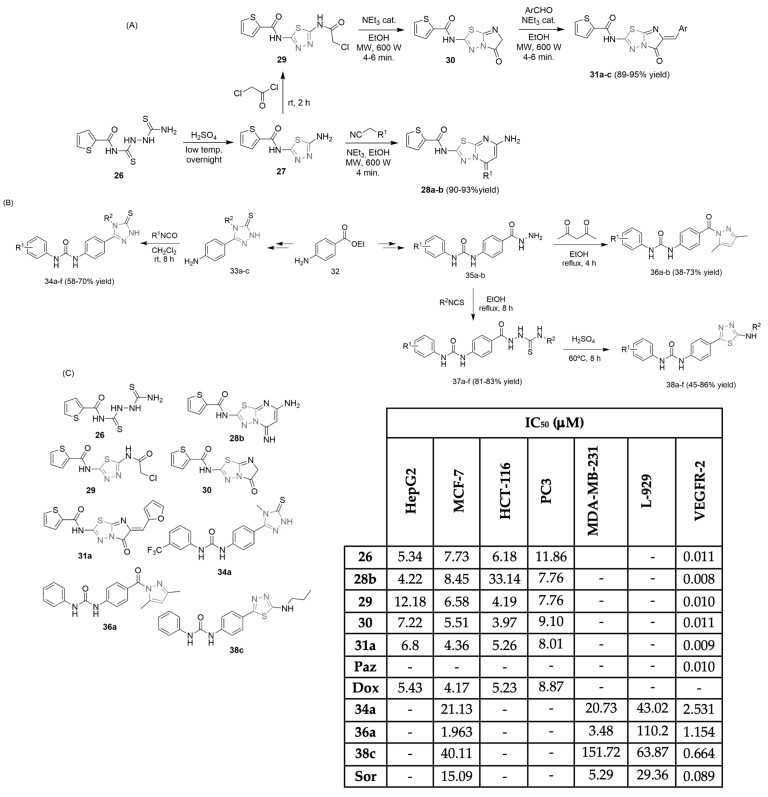
Synthesis of thiadiazole and other azole-hybrid scaffolds (**A**,**B**) and biological profiles (**C**) of the most promising ones. (HepG2: hepatic carcinoma cells; MCF-7 and MDA-MB-231: breast cancer cells; HCT-116: colon cancer cells; PC3: prostate cancer cells; L-929: non-cancerous fibroblast cells; Paz: pazopanib; Dox: doxorubicin; Sor: sorafenib).

Atta-Allah, AboulMagd, and Farag developed a green and efficient protocol to access 1,3,4-thiadiazole hybrids using microwave activation [22]. The general synthetic pathway (Scheme 4A) started with *N*-(2-carbamo-thioylhydrazine-1-carbonothioyl)thiophene-2-carboxamide **26**, which was left overnight at a low temperature in concentrated H_2_SO_4_, resulting in the formation of *N*-(5-amino- 1,3,4-thiadiazol-2-yl)thiophene-2-carboxamide **27** with a 90% yield. Using different carbon electrophilic species like ethyl cyanoacetate, malononitrile, and chloroacetyl chloride, it was possible to obtain compounds **28a**–**b** (under microwave conditions) and compound **29** (Scheme 4A). Moreover, 5-Oxo-imidazo-1,3,4-thiadiazole **30** was easily obtained from **29**, using microwave irradiation and ethanol as solvents. The aryl dihydroimidazo[2,1-b][1,3,4]thiadiazol-2-yl)thiophene-2-carboxamide derivatives **31a**–**c** were easily obtained under the Knoevenagel condensation reaction with several aldehyde precursors, using microwave irradiation (Scheme 4A). All new 1,3,4-thiadiazole derivatives were evaluated against four cancer cell lines: liver, breast, colon, and prostate (HepG2, MCF-7, HCT-116, and PC3, respectively), expressing moderate antiproliferative activities using doxorubicin as the reference control. Compounds **26**, **28b**, **29**, **30**, and **31a** were selected as the most promising ones, with IC_50_ values in the range of 3.97–33.14 μM (Scheme 4C). Also, the VEGFR-2 enzyme inhibition assay revealed potent inhibitory activity, with the 1,3,4-thiadiazole-based derivatives **28b** and **31a** being the most promising (IC_50_ values of 0.008 and 0.009 μM, respectively, to pazopanib’s 0.010 μM; see Scheme 4C). Docking studies on compound **28b** revealed the binding interactions with the VEGFR-2 enzyme, supporting the bioassays.

Danış, Küçükgüzelf, and co-workers described the synthesis of 1,3,4-thiadizoles and other azole–urea derivatives and evaluated their biological profiles as VEGFR-2 inhibitors (Scheme 4B,C). [23] Benzocaine **32** was used as a starting building block and the azole–urea derivatives were obtained via two synthetic routes (Scheme 4B). The 1,2,4-triazole–urea derivatives **34a**–**f** were obtained via cyclo-condensation, simultaneous deprotection of the benzoyl group, and final coupling reactions with isothiocyanates. The pyrazole–urea derivatives **36a**–**b** were obtained from the previously formed hydrazine derivative **35**, in reflux conditions with 2,4-dioxopentane. The thiadiazole–urea hybrids **38a**–**f** were obtained in a two-step reaction, first from hydrazine derivative **35** and finally from thiosemicarbazide intermediates **37a**–**f** (Scheme 4B). Screening the new derivatives for VEGFR-2 activity established compounds **34a**, **36a**, and **38c** as the most promising, with IC_50_ values of 2.531, 1.154, and 0.664 μM, respectively (Scheme 4C). Sorafenib (Figure 1) was used as a reference control. The antiproliferative evaluation was performed on the previously described compounds in two breast cancer cell lines (MCF-7 and MDA-MB-231) and in normal cells (L-929). Compound **36a** displayed the highest cytotoxic activity against MCF-7 and MDA-MB-231 breast cancer cells (with IC_50_ values of 1.963 and 3.48 μM, respectively; see Scheme 4C) and lower toxicity against non-tumor L-929 cells. The docking simulation with compound **38c**, the most potent regarding VEGFR-2 inhibition, explains the binding interactions.

**Scheme 5:**
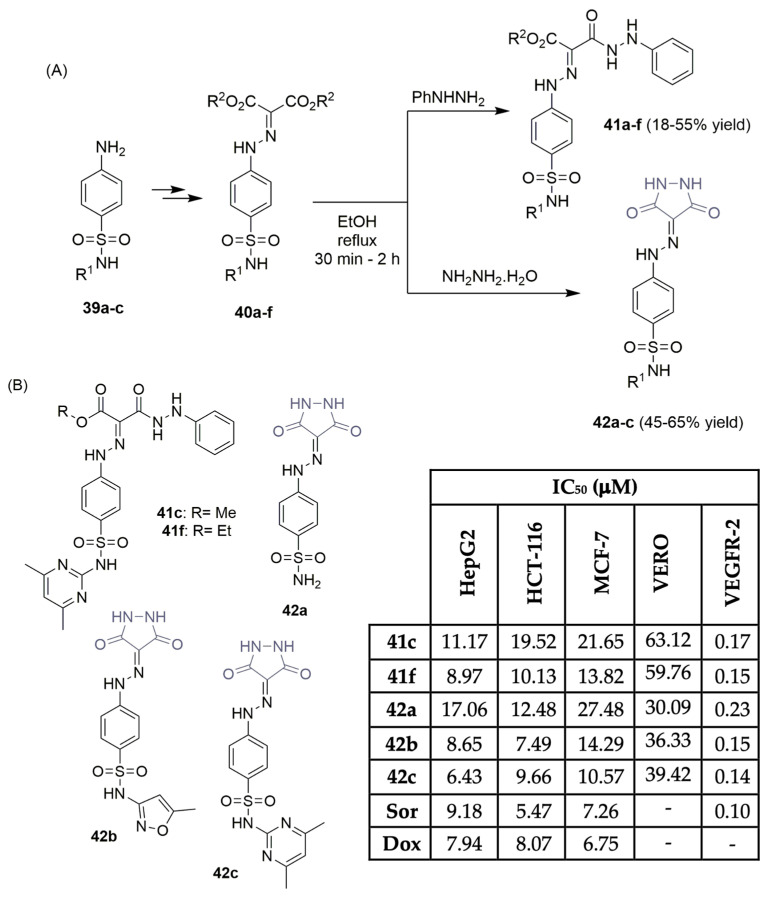
Synthesis (**A**) and anticancer evaluation (**B**) of 3,5-dioxopyrazolidine hybrids. (HepG2: hepatic carcinoma cells; HCT-116: colon cancer cells; MCF-7: breast cancer cells; VERO: normal kidney cells; Sor: sorafenib; Dox: doxorubicin).

El-Adl et al. described the synthesis of a small library of 3,5-dioxopyrazolidine derivatives and tested their behaviors as VEGFR-2 inhibitors (Scheme 5) [24]. Based on the molecular structure of sorafenib (Figure 1), and starting with aniline-derived sulfonamides **39a**–**c**, after a two-step reaction sequence, the corresponding diethyl or dimethyl 2-(2-(4-substitutedsulfamoylphenyl)-hydrazineylidene)malonate derivatives **40a**–**f** were obtained in moderate to excellent yields (Scheme 5A). Intermediates **40a**–**f** were treated with phenylhydrazine, in ethanol and reflux conditions, yielding the novel 4-((3,5-dioxopyrazolidin-4-yl) diazenyl)benzenesulfonamide derivatives **41a**–**f** in moderate yields. The same treatment with hydrazine hydrate enabled the formation of new 3,5-dioxopyrazolidin-4-yl derivatives **42a**–**c** in moderate yields (Scheme 5A). The antiproliferative activities of the new compounds were assessed against three human tumor cell lines: hepatocellular carcinoma, colorectal carcinoma, and breast cancer (HepG2, HCT-116, and MCF-7, respectively). The best results can be seen in Scheme 5B, where compounds **41c**, **41f**, and **42a**–**c** were determined to be the most potent, with IC_50_ values between 6.43 and 27.48 μM. Sorafenib (Figure 1) and doxorubicin were used as reference drugs, and curiously, compound **42c** displayed similar antiproliferative activity. Low toxicity values were found in the assay using VERO normal cells (IC_50_ values ranged from 30.09 to 63.12 μM; see Scheme 5B). The inhibitory activities of compounds **41c**, **41f**, and **42a**–**c** were determined against the VEGFR-2 enzyme, using sorafenib (Figure 1) as the reference control. All the compounds presented IC_50_ values between 0.23 and 0.14 μM, close to an IC_50_ of 0.10 μM (for sorafenib) (Scheme 5B), with compound **42c** being the most potent. Docking data highlighted the role of the diazene linker regarding affinity for the VEGFR-2 enzyme.

**Scheme 6:**
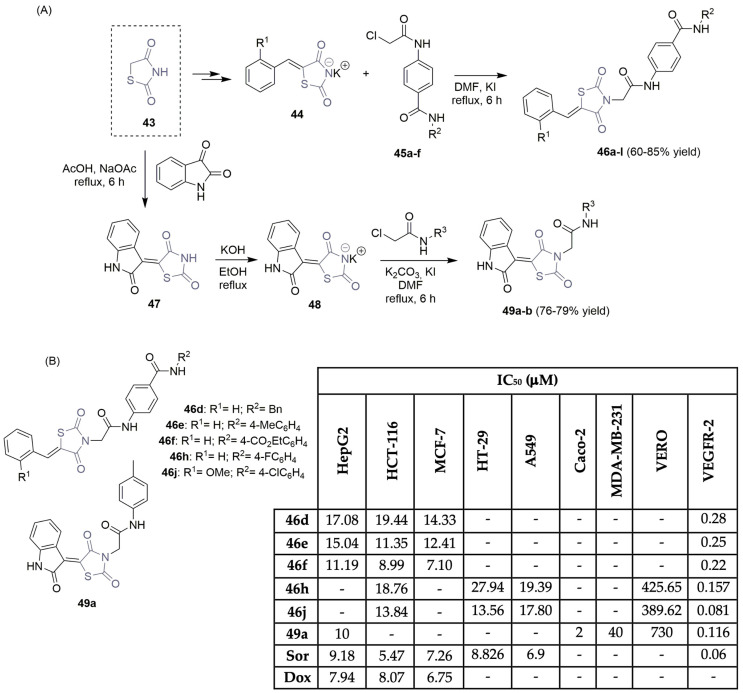
Synthesis (**A**) and anticancer evaluation (**B**) of thiazolidine-2,4-dione hybrids developed by Eissa’s group. (HepG2: hepatic carcinoma cells; HCT-116, HT-29 and Caco-2: colon cancer cells; MCF-7 and MDA-MB-231: breast cancer cells; A549: lung cancer cells; VERO: normal kidney cells; Sor: sorafenib; Dox: doxorubicin).

Eissa et al., motivated by the development of more potent VEGFR-2 inhibitors, described the synthesis and anticancer evaluation of new libraries of thiazolidine-2,4-dione derivatives (Scheme 6) [25,26,27]. Thiazolidine-2,4-dione **43** was used as the main precursor in a major synthetic plan, easily obtained through the cyclo-condensation reactions of thiourea and chloroacetic acid. In a two-step reaction approach, thiazolidine-2,4-dione **43** underwent Knoevenagel condensation with benzaldehyde derivatives, yielding 5-benzylidenethiazolidine-2,4-dione intermediates, which—under treatment with KOH—provided the corresponding potassium salts **44** (Scheme 6A). Refluxing those with appropriate intermediates **45a**–**f** led to the corresponding 5-benzylidenethiazolidine-2,4-dione derivatives **46a**–**l**, in moderate to good yields via an alkylation reaction [25,26]. Moreover, 2-oxoindoline-thiazolidine-2,4-dione hybrids **49a**–**b** were successfully obtained using a similar reaction protocol (Scheme 6A) [27]. The new thiazolidine-2,4-dione derivatives were tested for their antiproliferative activity against several human tumor cell lines, namely hepatic, colorectal, breast, and lung cancer cells (HepG2, HCT-116, and HT-29 and Caco-2, MCF-7 and MDA-MB-231, A549, respectively). The most potent and promising scaffolds can be found in Scheme 6B. Compounds **46f** and **49a** were found to be the most efficient at stopping the growth of several cancer cell lines. IC_50_ values between 7.10 and 11.19 μM were obtained for compound **46f** (HepG2, HCT-116, and MCF-7 cell lines) and 2–10 μM for compound **49a** (HepG2 and Caco-2 cell lines) (Scheme 6B). Also, **46j** (noted for its good profile in antiproliferative assays) and **49a** were found to be the most potent hybrids inhibiting VEGFR-2 at IC_50_ values of 0.081 μM and 0.116 μM, respectively. *In vitro* studies on VERO non-tumor cell lines were performed for some thiazolidine-2,4-dione derivatives, demonstrating a safety profile. Additional studies revealed that compound **46j** increased apoptosis in HT-29 cancer cells and details about the interaction between the inhibitor **46j** and the VEGFR-2 enzyme can be found [26].

#### 2.1.2. Five-Membered Ring Heterocycles with Benzo-Fused Aromatic Rings

Indazole is an example of this subclass of compounds that can be found in VEGFR-2 inhibitors used in clinical practice, namely in pazopanib and axitinib; therefore, it is often used in hybridization approaches in the quest for new bioactive compounds.

**Scheme 7:**
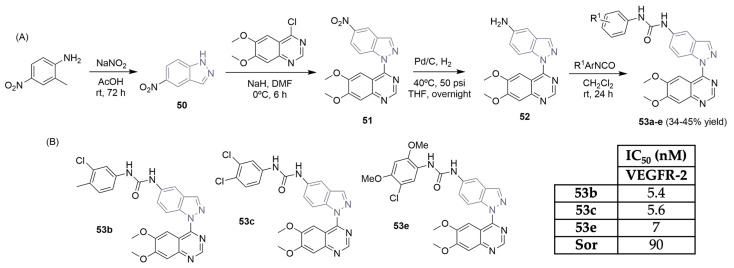
Synthesis (**A**) and VEGFR-2 activity profiles (**B**) of novel indazole-based derivatives. (Sor: sorafenib).

Abouzid and co-workers established the importance of indazole-based scaffolds as promising candidates for cancer treatment. The synthesis, antiangiogenic, and antiproliferative anticancer activities of new dimethoxyquinazoline-indazole-aryl-urea derivatives **53a**–**e** were described (Scheme 7) [28]. The diazotization reaction of 2-metyl-4-nitroaniline yielded 5-nitro-1*H*-indazole **50** in a 72% yield. The dimethoxyquinazoline-indazole intermediate **51** was easily obtained in the presence of NaH and at low temperatures. After the reduction of the nitro group in intermediate **51** using Pd/C and H_2_, amino-derivative **52** reacted with the corresponding aryl isocyanates, yielding the corresponding dimethoxyquinazoline-indazole-aryl-urea derivatives **53a**–**e** (Scheme 7A). These derivatives exhibited excellent activity profiles in the VEGFR-2 kinase inhibition assay (Scheme 7B). Compounds **53b**, **53c**, and **53e** were the most potent, with IC_50_ values of 5.4, 5.6, and 7 nM, displaying better results than sorafenib (Figure 1), the reference drug used in the assay (IC_50_ of 90 nm). The authors denoted the importance of having di-substitution or tri-substitution in the aromatic moiety linked to urea. Compounds **53b** and **53c** demonstrated strong inhibition of human umbilical vein endothelial cell (HUVEC) proliferation with 80 and 99.6% inhibition percentages at a 10 μM concentration, respectively. The most promising hybrid, **53c**, was also evaluated for its antiproliferative effect on a full panel of cancer lines, including, leukemia, non-small cell lung cancer, colon cancer, CNS cancer, melanoma, ovarian cancer, renal cancer, prostate cancer, and breast cancer, exhibiting a mean GI% of 130% and showing remarkable behaviors in terms of activity and safety, compared to several cell lines.

**Scheme 8:**
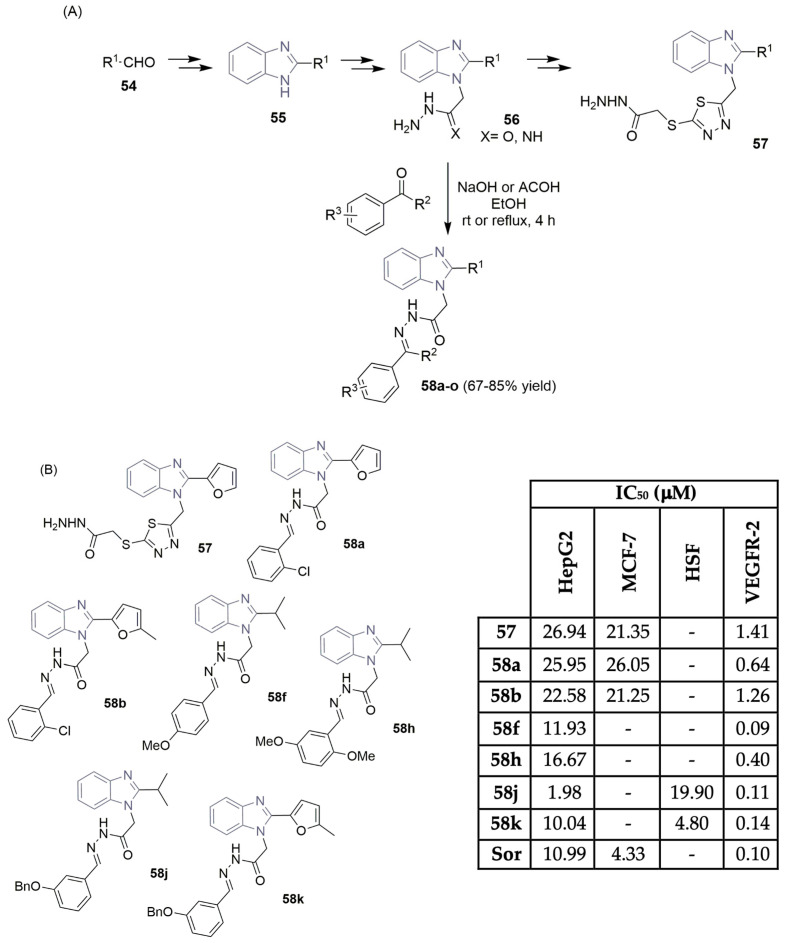
Synthesis (**A**) and bioprofile (**B**) of novel 1,2-disubstituted benzimidazole derivatives. (HepG2: hepatic carcinoma cells; MCF-7: breast cancer cells; HSF: normal skin cells; Sor: sorafenib).

Abdel-Mohsen et al. described the design and synthesis of 1,2-disubstituted benzimidazole derivatives in order to find efficient VEGFR-2 inhibitors (Scheme 8) [29,30]. The benzimidazole core was obtained in a two-step reaction approach, starting with the reaction of aldehyde derivatives **54** with Na_2_S_2_O_5_ to obtain the corresponding bisulfite adducts, which subsequently reacted with 1,2-phenylenediamine, yielding the desired 1-substituted-benzimidazole derivatives **55** (Scheme 8A). Multi-step reactions, including alkylation, hydrolysis, and reaction with hydrazine hydrate, led to the formation of the corresponding aceto-hydrazines or acetamido-hydrazines **56**. Condensation reactions with different aldehydes or ketones under basic or acid reaction conditions yielded Schiff bases **58a**–**o**, achieving moderate to good yields (Scheme 8A). The 2-fury benzimidazole **57** was obtained by alkylation reactions of the acetamido-hydrazine derivative **56** following conversion to the hydrazine derivative, in a two-step reaction approach. The new 1,2-disubstituted benzimidazole derivatives **57** and **58** were tested for their *in vitro* cytotoxicity against liver carcinoma cell line HepG2 and human breast cancer cell line MCF-7, using sorafenib (Figure 1) as the reference control (Scheme 8B). Benzimidazoles with 1-furyl and 1-isopropyl group demonstrated potent inhibitory activity in HepG2 cells, with compound **58j** being the most promising one (with an IC_50_ of 1.98 µM compared to sorafenib’s IC_50_ of 10.99 µM). The behavior of **58j** was assessed in normal human skin fibroblasts (HSFs), exhibiting higher selectivity to the HepG2 cell line compared to the HSF’s normal cell line (an IC_50_ of 19.90 µM compared to 1.98 µM; see Scheme 8B). Also, the most potent benzimidazoles were tested for their inhibition activities on the VEGFR-2 enzyme. Compounds **58f**, **58j**, and **58k** demonstrated promising VEGFR-2 inhibitory activities, with IC_50_ values of 0.09, 0.11, and 0.14 µM, respectively (very close to sorafenib, which had an IC_50_ of 0.1 µM) (Scheme 8B). Docking studies highlight the importance of the 2-substituted benzimidazole unit, lodged in the allosteric hydrophobic back pocket of VEGFR-2.

**Scheme 9:**
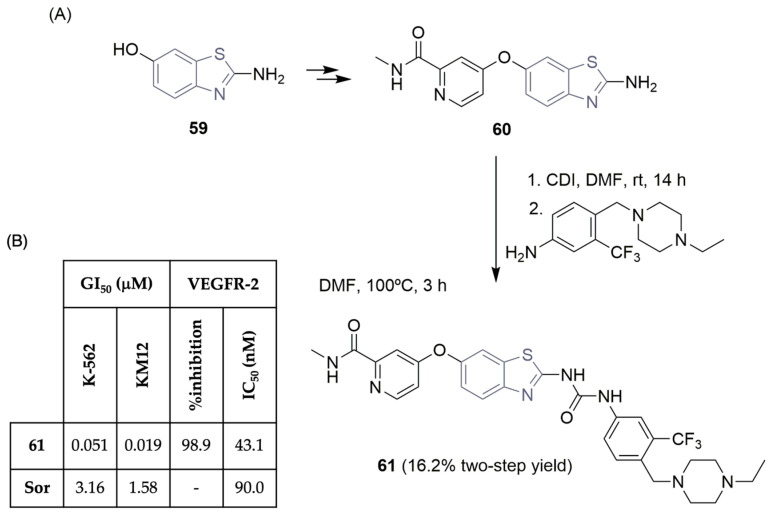
Synthesis (**A**) and bioprofile (**B**) of 4-((2-(3-(4-((4-ethylpiperazin-1-yl)methyl)-3-(trifluoromethyl)phenyl)ureido)benzo[d]thiazol-6-yl)oxy)picolinamide **61**. (K-562: leukemia cells; KM12: colorectal cancer cells; Sor: sorafenib).

Keum and co-workers worked on the rational design of scaffolds related to sorafenib (Figure 1) and found that urea-benzothiazole derivatives improved the inhibitory kinase activity and cellular potency [31]. Compound **61** demonstrated significant potency against most of the 60 human cancer cell lines, underlining GI_50_ values of 0.051 µM in leukemia K-562 cells and 0.019 µM in colorectal KM12 cells (Scheme 9B). Regarding VEGFR-2 inhibition, compound **61** displayed an IC_50_ value of 43.1 nM (against sorafenib’s 90 nM; see Scheme 9B). Additionally, compound **61** showed good activity in other oncogenic kinases, like Tie2, LCK, TrkA, wild-type, and T315I mutant ABL, and was considered by the authors as a promising multi-kinase inhibitor. Regarding the synthesis of compound **61**, two building blocks were used, the 2-aminobenzothiazole derivative **60** and the 4-((4-ethylpiperazin-1-yl)methyl)-3-(trifluoromethyl)aniline. Treatment of intermediate **60** with CDI (1,1′-carbonyldiimidazole) in DMF yielded the corresponding isocyanate, which reacted with the piperazine–aniline intermediate, providing the desired urea-benzothiazole **61** in a poor yield (16.2% two-step yield; see Scheme 9A). *In vivo*, the pharmacokinetic properties of compound **61** were evaluated, revealing a favorable profile with good oral bioavailability.

**Scheme 10:**
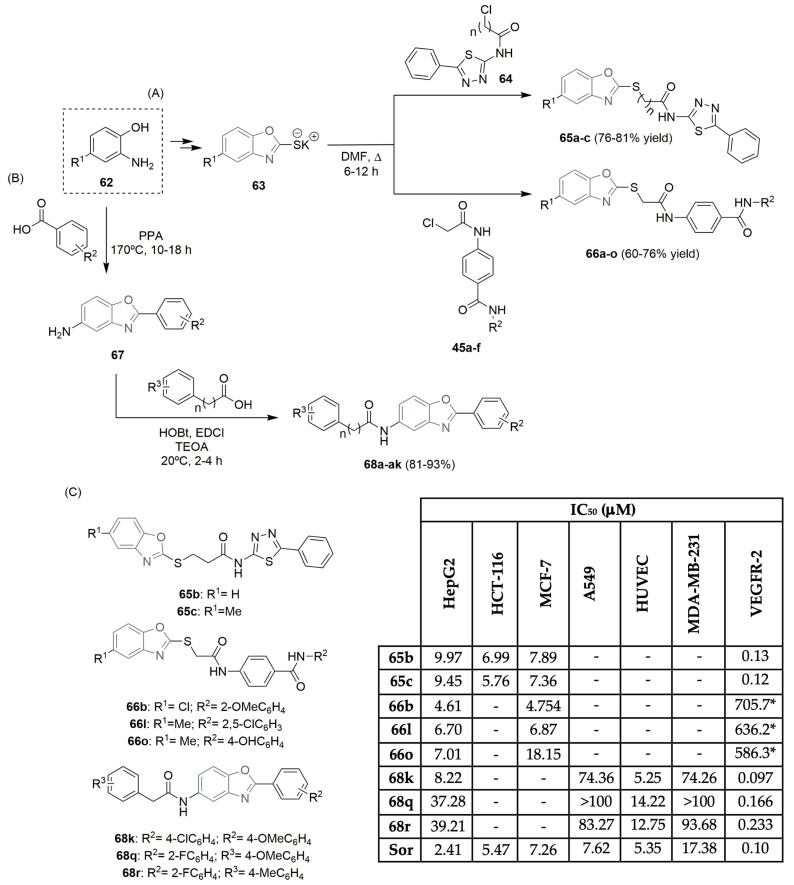
Synthesis (**A**,**B**) and bioprofile (**C**) of benzoxazole derivatives. * VEGFR-2 protein concentration (pg/mL) in HepG2 cells. (HepG2: hepatic carcinoma cells; HCT-116: colon cancer cells; MCF-7 and MDA-MB-231: breast cancer cells; A549: lung cancer cells; HUVEC: human umbilical vein endothelial cells; Sor: sorafenib; PPA: polyphosphoric acid; EDCI: 1-ethyl-3-(3-dimethylaminopropyl) carbodiimide; TEOA: triethanolamine).

Eissa et al. worked on designing new VEGFR-2 inhibitors based on five-membered ring heterocycles with benzo-fused aromatic rings; they reported interesting work regarded benzoxazole derivatives [32,33]. Considering previous work on thiazolidine-2,4-dione hybrids (Scheme 6), a new synthetic process was reported to access benzoxazole hybrids, starting with the 2-aminophenol derivative **62** (Scheme 10A). A two-step reaction approach, including the addition of carbon disulfide and treatment with alcoholic KOH, yielded the benzoxazole potassium salts **63**. Refluxing the appropriate potassium salts with chloroamide derivative **64** provided the corresponding benzoxazole/benzothiazole derivatives **65a**–**c**, achieving good yields (Scheme 10A). Using the same benzoxazole potassium salt **63** as a precursor, a new library of benzoxazoles **66a**–**o** was obtained in moderate to good yields, using benzo-chloroamide derivatives **45a**–**f** (Scheme 10A). Antiproliferative activities of the new benzoxazole derivatives **65** and **66** were evaluated against three tumor human cell lines, namely, hepatic, colon, and breast (HepG2, HCT-116, and MCF-7, respectively), with sorafenib (Figure 1) as a reference control. The results can be seen in Scheme 10C. Promising cytotoxic effects were found, particularly for compound **66b**, with IC_50_ values of 4.61 μM and 4.754 μM in HepG2 and MCF-7 cell lines, respectively. The evaluation of their *in vitro* effects on the VEGFR-2 enzyme demonstrated inhibitory potency with IC_50_ values nearly as similar to those of sorafenib (Scheme 10C).

Li, Lin, Zhang, Jin, and co-workers documented a new synthetic pathway to access other benzoxazole derivatives and tested their *in vitro* evaluation in cancer cells (Scheme 10B,C) [34]. Using the same 2-aminophenol precursor **62**, the 2-arylbenzoxazole intermediate **67** was obtained by reacting with different substituted benzoic acids in polyphosphoric acid at high temperatures. Amidation of intermediate **67** with substituted aromatic carboxylic acids using EDCI (1-ethyl-3-(3-dimethylaminopropyl)carbodiimide) and HOBt (hydroxybenzotriazole) as condensing agents, and TEOA (triethanolamine) as the base, led to the formation of a large library of 2-aryl benzoxazole derivatives **68**, achieving good yields (Scheme 10B). Their inhibitory activities were evaluated against normal cells (HUVEC) and three cancer cell lines (HepG2, A549, and MDA-MB-231) using sorafenib (Figure 1) as a positive control (Scheme 10C). Compound **68k** displayed the best results regarding cytotoxicity and showed high inhibitory activity against VEGFR-2 (IC_50_ of 0.097 μM) (Scheme 10C).

**Scheme 11:**
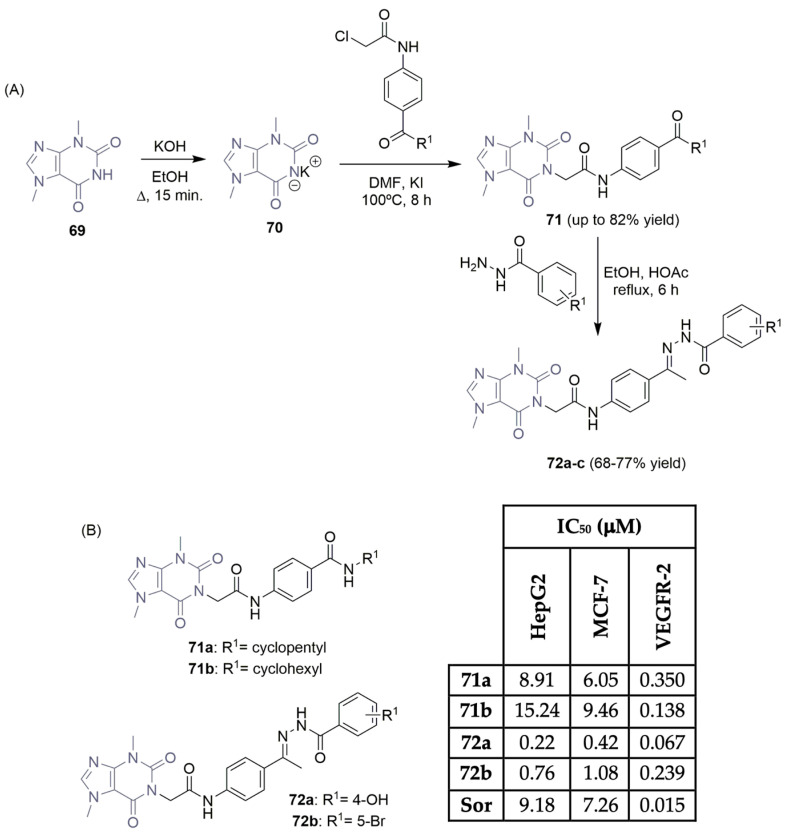
Synthesis (**A**) and bioprofile (**B**) of novel theobromine derivatives. (HepG2: hepatic carcinoma cells; MCF-7: breast cancer cells; Sor: sorafenib).

Theobromine, a natural product consisting of a pyrimidinedione, and an imidazole fused ring, is well known for its essential biological activities (e.g., it is a potential anti-cancer drug) [35]. Recently, Eissa et al. documented their latest discoveries regarding new theobromine derivatives inhibiting the VEGFR-2 enzyme, demonstrating promising antiproliferative activity in hepatic (HepG2) and breast (MCF-7) cancer cell lines (Scheme 11) [36,37,38]. Starting with the commercially available theobromine **69** as a building block, the corresponding potassium salt **70** was obtained by refluxing **69** with an alcoholic solution of KOH. Chloramide intermediates were used to synthesize the corresponding theobromine derivatives **71**, achieving good yields (Scheme 11A). Condensation of theobromine derivatives **71** with benzohydrazide intermediates (previously synthesized by refluxing the appropriate benzoate derivatives with hydrazine hydrate) provided the second family of new theobromine derivatives **72** (Scheme 11A). Scheme 11B summarizes the inhibitory activities of the most promising compounds in two cancer cell lines and the VEGFR-2 enzyme. Compound **72a** emerged as the most potent one, with remarkable activity against both cell lines (IC_50_ values of 0.22 μM and 0.42 μM for HepG2 and MCF-7, respectively). Additionally, compound **72a** demonstrated the best inhibitory activity in the VEGFR-2 enzyme (IC_50_ of 0.067 μM) (Scheme 11B). Additional studies disclosed that compound **72a** induced apoptosis in HepG2 cells, and docking simulations supported the binding interactions with the VEGFR-2 enzyme.

### 2.2. Six-Membered Ring Heterocycles

#### 2.2.1. Six-Membered Ring Heterocycles with One and Two Heteroatoms

The pyridine heterocycle is found in drugs such as sorafenib and regorafenib, which are examples of molecules that have reached the market or at least the clinical trial stage in the drug discovery pipeline, respectively.

**Scheme 12:**
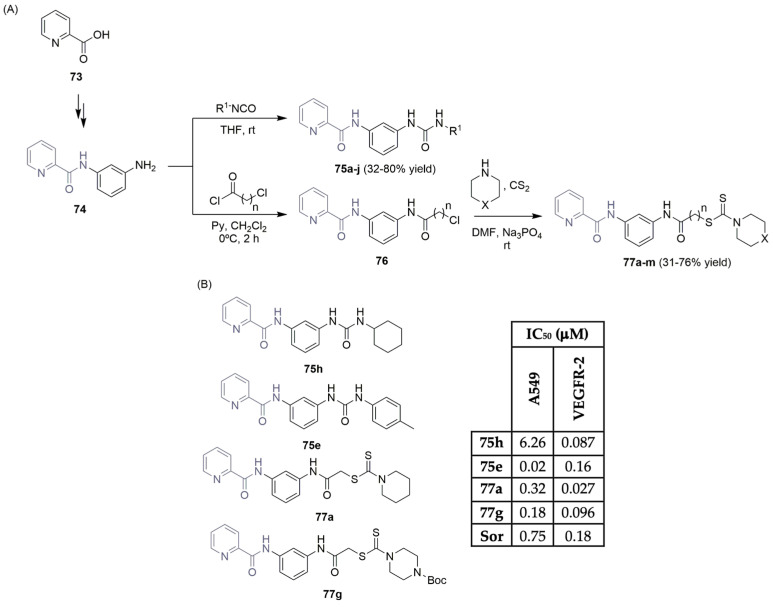
Synthesis (**A**) and bioprofile (**B**) of novel picolinamide derivatives. (A549: lung cancer cells; Sor: sorafenib).

Selim and co-workers described the synthetic pathways used to access small libraries of new picolinamide hybrids featuring (thio)urea and dithiocarbamate units and studied their behaviors as anticancer agents (Scheme 12) [39]. Using 2-picolinic acid **73** as the starting material, a two-step reaction sequence (coupling reaction followed by a reduction) yielded the aniline intermediate **74**. Treating intermediate **74** with iso(thio)isocyanates gave access to the picolinamide-(thio)urea derivatives **75** in moderate to good yields (Scheme 12A). On the other hand, a base-mediated acylation reaction of **74** with chloroacetyl- or chloropropionyl chloride, followed by a reaction with appropriate cyclic secondary amines, led to the easy synthesis of picolinamide-dithiocarbamate derivatives **77** in moderate to good yields (Scheme 12A). The new libraries were evaluated for their cytotoxic activity against a lung cell cancer line (A549) and for their VEGFR-2 inhibitory activity. The most promising compounds can be seen in Scheme 12B. Compound **75e** showed the best IC_50_ value against A549 (0.02 μM), whereas compound **77a** demonstrated potent inhibitory activity against VEGFR-2 (IC_50_ of 0.027 μM), compared to sorafenib (Figure 1), the reference drug. Compound **77a** was docked into the VEGFR-2 binding site, demonstrating similar binding interactions as sorafenib.

**Scheme 13:**
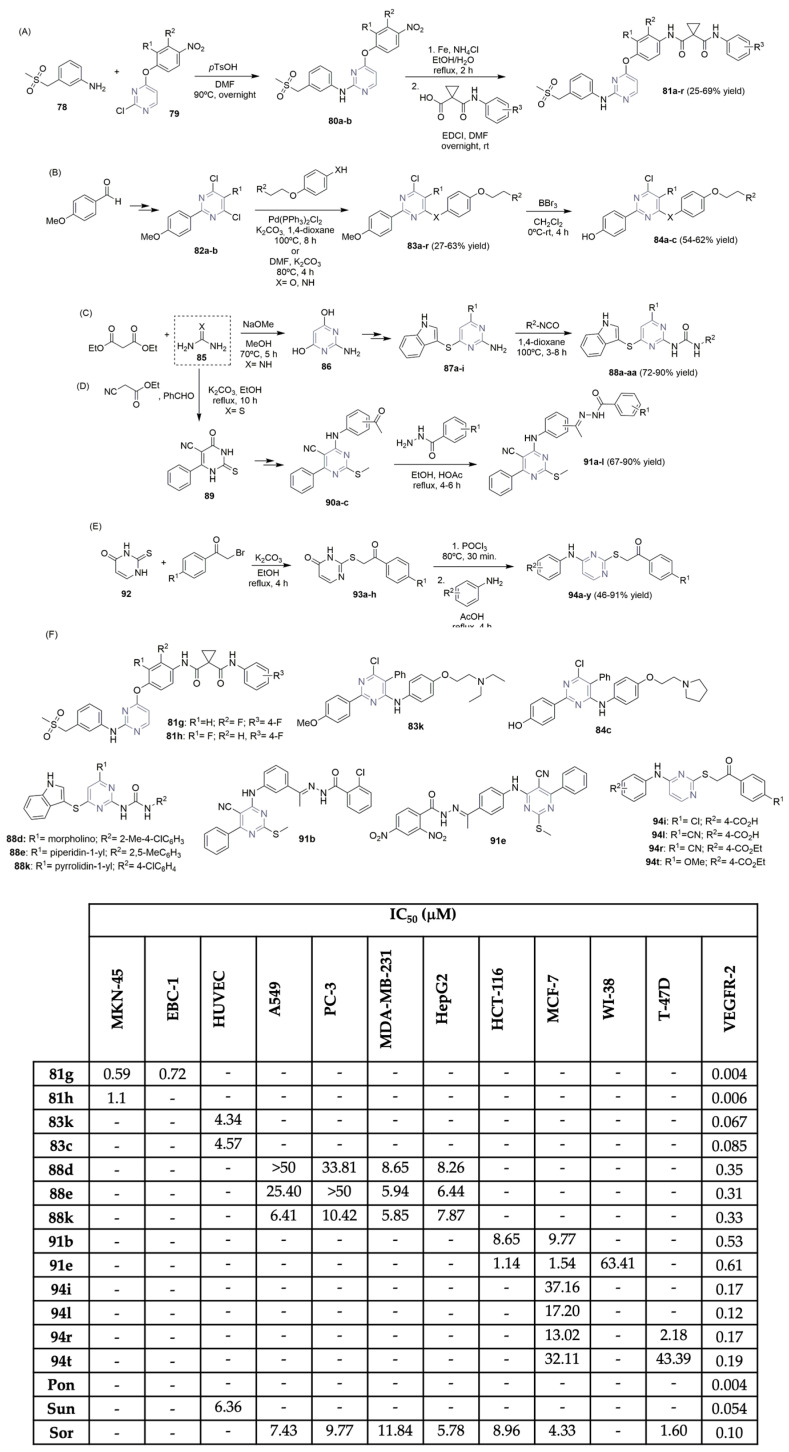
Synthesis (**A**–**E**) and bioprofile (**F**) of novel pyrimidine derivatives. (MKN-45: gastric carcinoma cells; HUVEC: human umbilical vein endothelial cells; EBC-1 and A549: lung cancer cells; PC-3: prostate cancer cells; MDA-MB-231, MCF-7 and T-47D: breast cancer cells; Hep-G2: liver cancer cells; HCT-116: colon cancer cells; WI-38: normal human fibroblast cells; Pon: ponatinib; Sun: sunitinib; Sor: sorafenib).

Pyrimidine derivatives demonstrated to be powerful units in the search for VEGFR-2 inhibitors, being present in the chemical structure of pazopanib. Xu, Geng, Duan, and co-workers described the synthesis and biological profiles (Scheme 13A,F) of new anilinopyrimidines as dual kinase inhibitors, VEGFR-2 and c-Met [40]. Intermediates **80a**–**b** were easily obtained using an acid-catalyzed reaction of chloro-pyrimidines **79** with aniline **78.** After a reduction reaction with iron and condensation with the corresponding carboxylic acid derivatives, the new anilinopyrimidine derivatives **81a**–**r** were obtained in moderate yields (Scheme 13A). The library was evaluated regarding the VEGFR-2 activity and an interesting SAR study was conducted to verify the best substituents in the aromatic rings of **81**. Compounds **81g** and **81h**, with fluorine groups substituted in the aromatic rings, demonstrated potent inhibitory activities in VEGFR-2 (0.004 μM and 0.006 μM, respectively), gastric carcinoma cells (MKN-45), and lung cancer cells (EBC-1) (Scheme 13F). The X-ray structure of **81h** showed that this compound engages the ATP-binding site. A few years later, Xiang et al. described their findings regarding new 2,4-disubstituted pyrimidines with anti-breast cancer activity (Scheme 13B,F) [41]. Moreover, 4-methoxybenzaldehyde was used as the starting material to access pyrimidine intermediates **82a**–**b** in a three-step sequence, which were then subjected to combinations of different side chains (acetoamidoanilines or 4-ethoxianilines), yielding the target 2,4-disubstituted pyrimidine compounds **83a**–**r** in moderate yields. The corresponding hydroxy-derivatives **84a**–**c** were easily obtained via a demethylation reaction with BBr_3_ (Scheme 13B). The evaluation of certain compounds for their inhibition activity against VEGFR-2 and antiproliferative activity toward VEGFR-2 overexpressed human umbilical vein endothelial cells (HUVECs) was reported, using sunitinib (Figure 1) used as the positive control (Scheme 13F). Compounds **83k** and **84c** displayed the best activity values in the VEGFR-2 assay (IC_50_ values of 0.067 μM and 0.085 μM, respectively). Recently, Shankaraiah and co-workers used the molecular hybridization strategy to combine two important pharmacophoric units (pyrimidine and thioindole) into a single element, developing a new family of pyrimidine–thioindole hybrids as potent VEGFR-2 inhibitors (Scheme 13C,F) [42]. The condensation reaction between diethyl malonate and guanidine hydrochloride **85** leads to the formation of the 2-aminopyrimidine-4,6-diol derivative **86**. A two-step reaction approach (double chlorination followed by nucleophilic substitution with aromatic amines, cyclic non-aromatic amines, or indole-3-thiol) yielded the intermediates **87a**–**i**. A library of carbamide derivatives of thioether-linked indole-pyrimidine compounds **88a**–**aa** was synthesized in good yield by reacting intermediates **87a**–**i** with substituted aryl isocyanates (Scheme 13C). An evaluation of their *in vitro* cytotoxicity against lung, prostate, breast, and liver cancer cell lines (A549, PC-3, MDA-MB-231, and HepG2, respectively) revealed moderate to significant antiproliferative activity (Scheme 13F). Compound **88k** demonstrated the best cytotoxic profile for the assayed cancer cell lines (IC_50_ between 5.85 and 10.42 μM). Compounds **88d**, **88e**, and **88k** exhibited strong inhibitory activity regarding the VEGFR-2 enzyme (Scheme 13F), with IC_50_ values between 0.31 and 0.35 μM, in contrast to 0.21 μM for sorafenib (Figure 1), the positive control. Recently, Khalifa, Eissa and co-workers described the synthesis of a new library of pyrimidine-5-carbonitrile derivatives using 2-mercapto-6-oxo-4-phenyl-1,6-dihydropyrimidine-5-carbonitril **89** as the intermediate, via cyclocondensation reaction between thiourea **85**, benzaldehyde, and ethyl cyanoacetate, in basic conditions (Scheme 13D) [43]. The corresponding target intermediates **90a**–**c** were obtained through a three-step sequence involving alkylation, chlorination, and amination with aromatic amines. Condensation reaction with acid hydrazide derivatives produced the corresponding pyrimidine-5-carbonitrile derivatives **91a**–**l** in moderate to good yields (Scheme 13D). *In vitro* cytotoxicity was evaluated against colon and breast cancer cell lines (HCT-116 and MCF-7, respectively) and the most promising compounds can be seen in Scheme 13F. Pyrimidine derivatives **91b** and **91e** displayed higher cytotoxic activities against the HCT-116 and MCF-7 cell lines (IC_50_ ranging from 1.14 to 9.77 μM). Compound **91e** revealed cytotoxic IC_50_ values of 63.41 μM compared to normal human lung cells (WI-38; see Scheme 13F), much lower against the cancer cells. The *in vitro* assay for VEGFR-2 inhibitory activity was conducted on the most promising compounds, demonstrating very good inhibitory activity for compounds **91b** and **91e** (IC_50_ values of 0.53 μM and 0.61 μM, respectively), compared with that of sorafenib (IC_50_ of 0.19 μM) (Figure 1), the positive control (Scheme 13F). Taking into account the work reported so far on pyrimidine derivatives, Abdel-Mohsen et al. discovered the potential of substituted 4-amino-2-thiopyrimidines as VEGFR-2 inhibitors (Scheme 13E,F) [44]. Moreover, 2-thiouracil **92** was the starting material, together with bromoacetophenone, in basic media, accessing the intermediates **93a**–**h**. After chlorination with POCl_3_, the desired 4-amine-2-thiopyrimidines **94a**–**y** were obtained by reacting **93a**–**h** with primary amines in acidic reflux conditions (Scheme 13E). This new library of 4-amine-2-thiopyrimidines **94a**–**y** was tested regarding VEGFR-2 inhibitory activity and cytotoxicity against breast cancer cell lines MCF-7 and T-47D (Scheme 13F). Compounds **94i**, **94l**, **94r**, and **94t** were the most potent (with IC_50_ in the VEGFR-2 assay of 0.17, 0.12, 0.17, and 0.19 μM, respectively), very close to that of sorafenib (0.10 μ); see Figure 1. Regarding antiproliferative assays on breast cancer cell lines, compound **94r** exhibited the best IC_50_ value (13.02 μM in MCF-7 cells and 2.18 μM in T-47D cells). Molecular docking reinforces the key binding interactions of the most promising compounds with the VEGFR-2 enzyme.

**Scheme 14:**
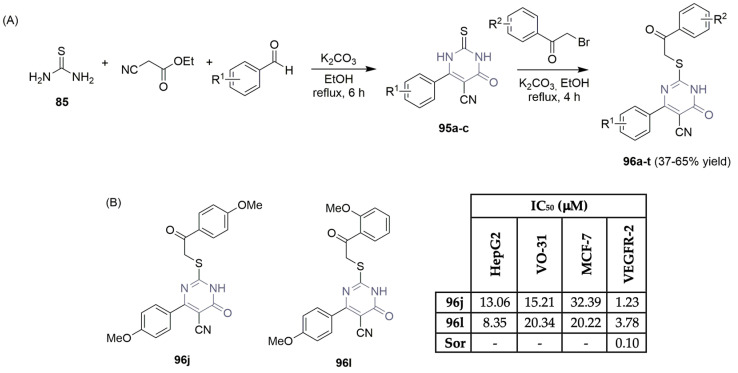
Synthesis (**A**) and bioprofile (**B**) of novel pyrimidinone derivatives. (Hep-G2: liver cancer cells; VO-31: renal cancer cells; MCF-7: breast cancer cells; Sor: sorafenib).

Abdel-Mohsen et al., motivated by noteworthy VEGFR-2 inhibitory activity obtained for 4-amine-2-thiopyrimidines **94a**–**y** (Scheme 13F), described the synthesis and bio-evaluation of a second new family of derivatives, with the pyrimidinone central unit (Scheme 14) [45]. The 2-thioxopyrimidinones **96a**–**t** were synthesized in a two-step reaction sequence, using thiourea **85**, ethyl cyanoacetate, and benzaldehyde derivatives as starting materials (Scheme 14A). Further reaction of intermediates **95a**–**c** with several 2-bromoacetophenone derivatives under basic reaction conditions yielded the corresponding 2-thioxopyrimidinones **96a**–**t**, achieving a moderate yield (Scheme 14A). The library was further evaluated regarding the inhibition of VEGFR-2 and antiproliferative activity on liver, renal, and breast cancer cells (HepG2, VO-31, and MCF-7, respectively; see Scheme 14B). Compounds **96j** and **96l** demonstrated moderate VEGFR-2 inhibitory activity as well as attractive antiproliferative activity on the screened cell lines (Scheme 14B). Despite the good bioprofile, the first library of compounds reported by these authors (Scheme 13E), which considered pyrimidine derivatives **94a**–**y**, featured better inhibition values against the VEGFR-2 enzyme and MCF-7 breast cancer cells (compare scaffolds **94i**, **94l**, **94r**, and **94t** in Scheme 13E and scaffolds **96j** and **96l** in Scheme 14B), demonstrating poor efficiency of the pyrimidinone central unit.

**Scheme 15:**
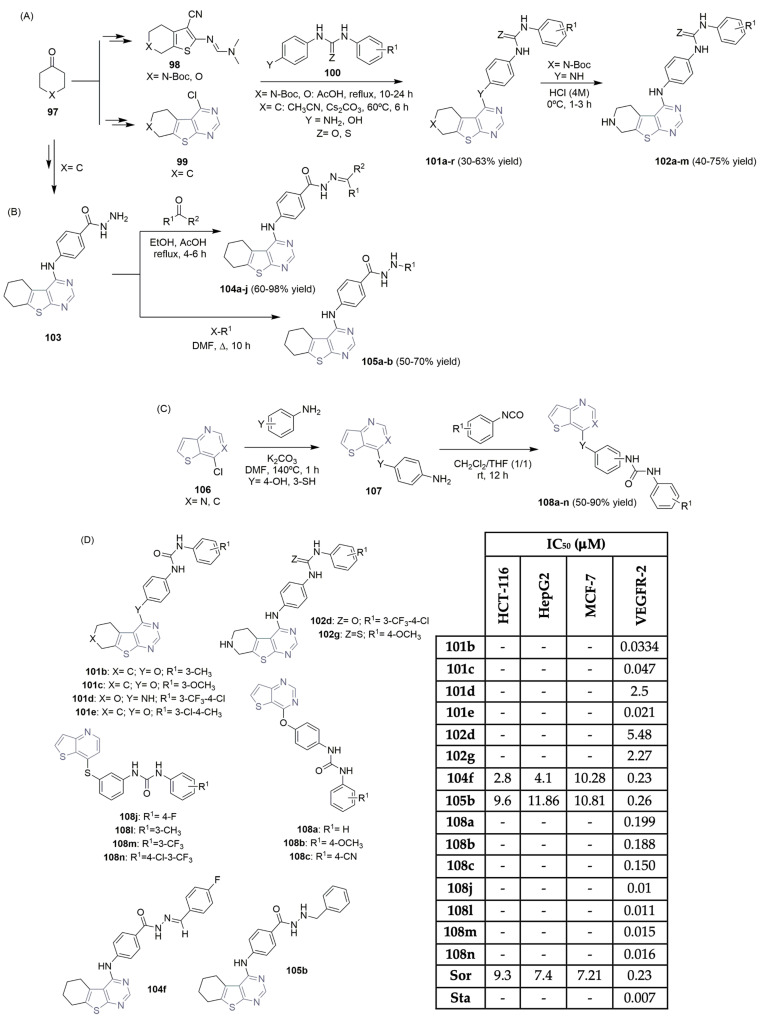
Synthesis (**A**–**C**) and bioprofile (**D**) of novel thieno-fused-pyrimidine and thieno-fused-pyridin derivatives. (HCT-116: colon cancer cells; Hep-G2: liver cancer cells; MCF-7: breast cancer cells; Sor: sorafenib; Sta: staurosporine).

Several research groups have described the potential of thieno-fused-pyrimidine scaffolds as cancer-targeting VEGFR-2 agents. Abouzid et al. described the synthesis and VEGFR-2 activity of new libraries of thieno[2,3-*d*]pyrimidines **101** and **102** (Scheme 15A,D) [46,47]. Using a cyclohexanone derivative as the starting material, intermediates **98** and **99** were obtained via a multi-step synthesis. The corresponding aromatic urea and thiourea derivatives were prepared easily from aniline derivatives and isocyanates, and intermediates **98** and **99** yielded the desired thieno[2,3-*d*]pyrimidines **101** in moderate yields (Scheme 15A). Deprotection of the Boc group under acidic reaction conditions provided the corresponding thieno[2,3-*d*]pyrimidines **102** in moderate to good yields (Scheme 15A). Thieno[2,3-*d*]pyrimidines **101b**, **101c**, and **101e**, linked to biarylurea via the ether linker, revealed highly potent nanomolar VEGFR-2 inhibition (IC_50_ values of 0.0334 μM, 0.047 μM, and 0.021 μM, respectively; see Scheme 15D). Molecular docking studies supported the results, revealing the ability of urea-based derivatives to bind to the VEGFR-2 enzyme. Eissa et al.—authors who are very active in the field—also described their discoveries regarding thieno[2,3-d]pyrimidine-based derivatives as potent VEGFR-2 inhibitors [48]. The hydrazine derivative intermediate **103** was easily obtained in a multi-step reaction sequence, starting with cyclohexanone and malononitrile. Reaction with several aldehydes or ketones led to the target Schiff’s bases **104a**–**j**, in moderate to excellent yields (Scheme 15B). Alkylation of hydrazine intermediate **103** with several alkyl halides resulted in the formation of thieno[2,3-*d*]pyrimidine derivatives **105a**–**b**, in moderate to good yields (Scheme 15B). The new library of thieno[2,3-*d*]pyrimidine derivatives **104** and **105** were evaluated regarded their antiproliferative activity against three human cancer cell lines, colon (HCT-116), liver (HepG2) and breast (MCF-7) cells (Scheme 15D), with sorafenib (Figure 1) as the positive control. The highest potency was recognized for compound **104f**, with IC_50_ values of 2.80 μM, 4.10 μM and 10.28 μM, respectively. Regarding VEGFR-2 activity, both compounds **104f** and **105b** showed the highest potency with IC_50_ values of 0.23 μM and 0.26 μM, very close to sorafenib at 0.23 μM (Scheme 15D). Queiroz and co-workers reported interesting work regarded the VEGFR-2 activity of new thieno [3,2-*d*]pyridine and pyrimidine derivatives **108** (Scheme 15C) [49,50,51]. Commercially available 7-hydroxythieno[3,2-*b*]pyridine or 7-hydroxythieno[3,2-*b*]pyrimidine was used as starting material. Chlorination with POCl_3_ yielded the intermediate **106**, which underwent nucleophilic aromatic substitution with aniline derivatives to access the aminated di(hetero)aryl intermediates **107**. The target aryl-thieno[3,2-*d*]pyridine or pyrimidine-phenyl ureas **108a**–**n** were easily obtained in 50–90% yield by reacting **107** with different substituted aryl isocyanates (Scheme 15C). The evaluation of the new libraries of **108** for their ability to interact with the VEGFR-2 kinase enzyme demonstrated that thieno[3,2-*b*]pyridine compounds **108j**, **108l**, **108m**, and **108n** were the most promising, showing IC_50_ values in the range of 0.01–0.016 μM (Scheme 15D), using staurosporine as the positive control. The presence of hydrophobic groups like CF_3_, F, and Cl in the terminal phenyl ring, together with an *S*-linker and the arylurea unit in the *meta* position, proved to be the best substitution pattern to access good inhibitory levels on the VEGFR-2 assay.

**Scheme 16:**
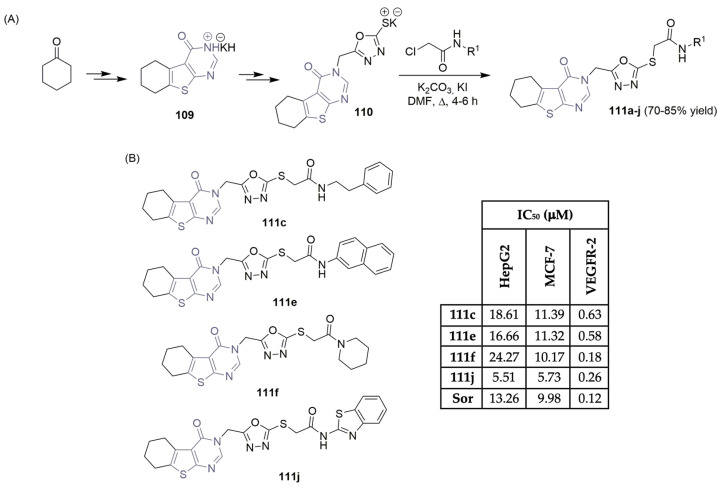
Synthesis (**A**) and bioprofile (**B**) of novel thieno[2,3-*d*]-pyrimidinone derivatives. (Hep-G2: liver cancer cells; MCF-7: breast cancer cells; Sor: sorafenib).

Eissa et al. described the design, synthesis, and antiproliferative evaluation of a new small library of thieno[2,3-*d*]pyrimidinone derivatives, targeting VEGFR-2 (Scheme 16) [52,53]. Using cyclohexanone and malononitrile as starting materials, the potassium salt intermediate **109** was formed in a three-step reaction sequence. Reaction with the appropriate acetamides, in a DMF/KI mixture, produced the target thieno[2,3-*d*]pyrimidinone derivatives **111a**–**j**, achieving good yields (Scheme 16A). Antiproliferative activities against liver and breast cancer cell lines demonstrated potent cytotoxic effects, with IC_50_ values ranging from 5.51 to 24.27 μM, using sorafenib (Figure 1) as the positive control (Scheme 16B). The assessment of inhibitory effects on VEGFR-2 revealed that compounds **111f** and **111j** exhibited good *in vitro* abilities with IC_50_ values of 0.18 μM and 0.26 μM, respectively, with sorafenib as the reference drug (with an IC_50_ value of 0.12 μM) (Scheme 16B).

**Scheme 17:**
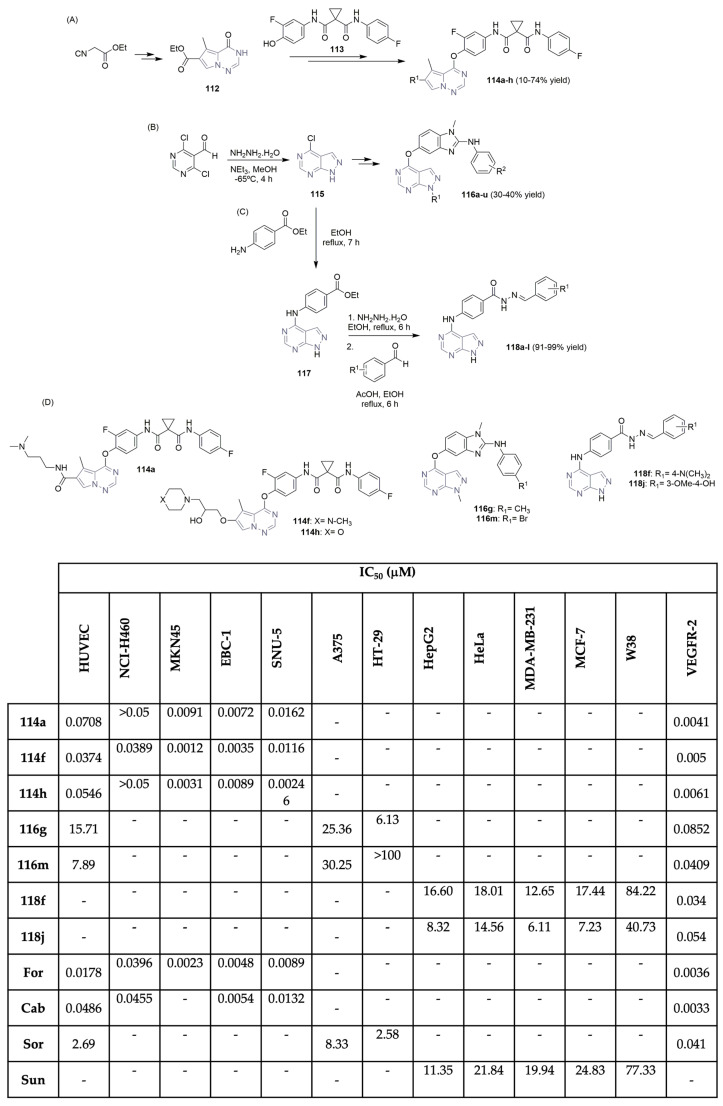
Synthesis of novel pyrrolo[2,1-f][1,2,4]triazine (**A**), 1*H*-pyrazolo[3,4-*d*]pyrimidine (**B**) and pyrrolo[2,3-*d*]pyrimidine (**C**) derivatives and bioprofile (**D**). (HUVEC: human umbilical vein endothelial cells; NCI-H460 and EBC-1: lung cancer cells; MKN45 and SNU-5: gastric cancer cells; A375: melanoma cells; HT-29: colorectal cancer cells; Hep-G2: liver cancer cells; HeLa: cervical cancer cells; MDA-MB-231 and MCF-7: breast cancer cells; W38: human fetal lung fibroblast cells; For: foretinib; Cab: cabozantinib; Sor: sorafenib; Sun: sunitinib).

To develop potent VEGFR-2 inhibitors, Huang, Qian, and co-workers synthesized a library of pyrrolo[2,1-*f*][1,2,4]triazine derivatives and evaluated their anticancer efficacy (Scheme 17A,D) [54]. Using ethyl 2-isocyanoacetate as the starting material, the intermediate ethyl 5-methyl-4-oxo-3,4-dihydropyrrolo[2,1-*f*][1,2,4]triazine-6-carboxylate **112** was easily obtained in a two-step reaction approach. After a multi-step reaction protocol, evolving group protection/deprotection, chlorination, Grignard, and Bayer–Villiger oxidation reactions, among others, the target pyrrolo[2,1-*f*][1,2,4]triazine derivatives **114a**–**h** were successfully obtained (Scheme 17A). Antiproliferative assays were performed for the library of pyrrolo[2,1-*f*][1,2,4]triazine derivatives **114**, against lung (NCI-H460 and BBC-1) and gastric (MKN45 and SNU-5) cancer cell lines using foretinib and cabozantinib as positive controls (Scheme 17D). Compounds **114a**, **114f**, and **114h** demonstrated nanomolar activity in the gastric cell line MKN45 and in the lung cancer cell line EBC-1, with IC_50_ values ranging from 0.0012 to 0.0091 μM. Compound **114a** showed the highest inhibitory effect on the VEGFR-2 enzyme, with an IC_50_ of 0.0041 μM, close to the positive controls foretinib and cabozantinib (IC_50_ values of 0.0036 μM and 0.0033 μM, respectively) (Scheme 17D). Zhang, Wu, and co-workers, aiming to explore novel VEGFR-2 inhibitors, reported novel 1*H*-pyrazolo[3,4-*d*]pyrimidine derivatives and studied their biological profile (Scheme 17B,D) [55]. Using the commercially available 4,6-dichloropyrimidine-5-carbaldehyde as the starting material, intermediate **115** was easily obtained using hydrazine hydrate, in methanol, at low temperatures (Scheme 17B). A multi-step series of reactions comprising S_N_2 reaction, S_N_Ar reaction, group protection, methylation, and reduction, among others, yielded the target 1*H*-pyrazolo[3,4-*d*]pyrimidine derivatives **116a**–**u**, in moderate yields. The **116a**–**u** library was evaluated for its inhibitory activities against human cancer A375 (melanoma) and HT-29 (colorectal) cell lines and VEGFR-2 kinase, using sorafenib (Figure 1) as the positive control (Scheme 17D). Compounds were also evaluated against the HUVEC cell line, which expresses the VGFR2-2 protein. The 1*H*-Pyrazolo[3,4-*d*]pyrimidine derivative **116m** exhibits the best inhibitory activity against VEGFR-2 (IC_50_ of 0.0409 μM), similar to the one expressed by sorafenib (IC_50_ of 0.041 μM). In particular, compound **116g** also showed potent antiproliferative activity against the colorectal cancer cell line HT-29, with an IC_50_ value of 6.13 μM, close to the one expressed by sorafenib, the positive control (IC_50_ of 2.58 μM) (Scheme 17D). Recently Rahman, Alanazi, and co-workers described the synthesis of a library of pyrrolo[2,3-*d*]pyrimidine derivatives and evaluated their antiproliferative activity against several cancer cell lines and VEGFR-2 selectivity (Scheme 17C,D) [56]. Moreover, 4-chloro-7*H*-pyrrolo[2,3-*d*]pyrimidine **115** was refluxed with ethyl-4-aminobenzoate in ethanol, yielding the ethyl-4-((7*H*-pyrrolo [2,3-*d*]pyrimidin-4-yl)amino) benzoate intermediate **117**. Reaction with hydrazine hydrate, followed by condensation with several benzaldehyde derivatives, yielded the target pyrrolo[2,3-*d*]pyrimidine derivatives **118a**–**l** in excellent yields (Scheme 17C). Evaluation of their cytotoxic effects against breast cancer cell lines MCF-7 and MDA-MB-231, hepatocellular carcinoma cell line HepG2, and epithelioid cervix carcinoma cell line HeLa, using sunitinib (Figure 1) as the positive control demonstrated variable levels of cytotoxic effects. Compounds **118f** and **118j** demonstrated the best antiproliferative activities against the tested cancer cell lines, with IC_50_ values ranging from 6.11 to 18.01 μM (Scheme 17D). Regarding VEGFR-2 activity, compound **118f** demonstrated the best profile, with an IC_50_ value of 0.034 μM, which was slightly more potent than sorafenib, which was used as the positive control (IC_50_ of 0.041 μM). Compounds **118f** and **118j** also revealed excellent selectivity against the tested cancer lines when compared with the IC_50_ values evaluated against the W38 normal cell line (human fibroblast).

**Scheme 18:**
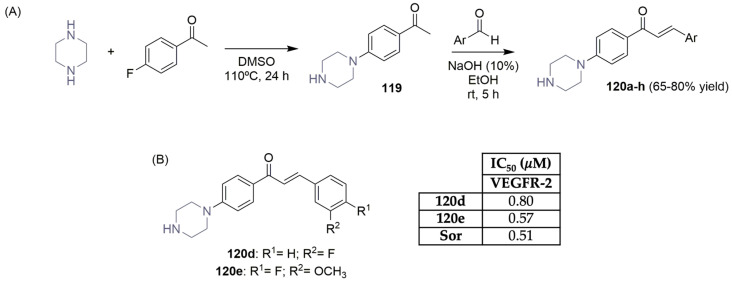
Synthesis (**A**) and VEGFR-2 activity (**B**) of novel piperazine–chalcone derivatives. (Sor: sorafenib).

Ahmed, Santali, and El-Haggar described the synthesis and VEGFR-2 evaluation of a novel small library of piperazine–chalcone hybrids (Scheme 18) [57]. Piperazine and 1-(4-fluorophenyl)ethan-1-one were used as starting material to obtain the piperazine-acetophenone intermediate **119**, which underwent chalcone formation with several benzaldehyde derivatives, achieving good yields, the target piperazine–chalcone hybrids **120a**–**h** (Scheme 18A). Selected derivatives were tested *in vitro* against a panel of 60 human cancer cell lines by NCI (Bethesda, Montgomery County, MD, US), at a single dose of 10 μM. Preliminary results showed promising cytotoxicity toward a variety of cancer cell lines, particularly for compounds **120d** and **120e**. *In vitro* evaluation of the ability of previously selected piperazine–chalcone hybrids **120a**–**h** revealed that compound **120e** was the most potent VEGFR-2 inhibitor, with an IC_50_ value of 0.57 μM, which was very close to sorafenib (Figure 1), the positive control (IC_50_ of 0.51 μM) (Scheme 18B).

#### 2.2.2. Six-Membered Ring Heterocycles with Benzo-Fused Aromatic Rings

**Figure 2:**
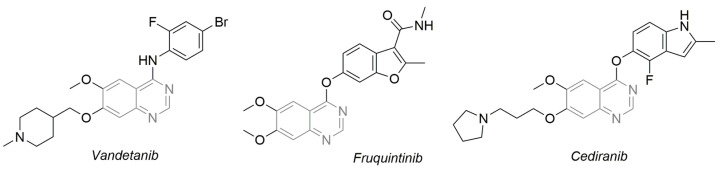
Examples of drugs used in clinical practice with the quinazoline unit.

The quinazoline unit, a nitrogen-containing aromatic bicyclic heterocycle consisting of two fused six-membered rings (and described as the first established scaffold in the evolution of kinase inhibitors), is a favored and flexible core with more than twenty approved drugs by the US Food and Drug Administration to date. Examples (Figure 2) include the first drug to treat late-stage medullary thyroid cancer (metastatic) in adult patients ineligible for surgery, the Astra Zeneca drug vandetanib (Caprelsa^®^), which was approved in 2011; fruquintinib (Elunate^®^ marketed in China by Hutchmed), which was approved for the treatment of metastatic colorectal cancer in 2018; and cediranib (AZD-2171), a highly potent VEGFR-2 inhibitor developed by Astra Zeneca (still in phase III clinical trials for ovarian cancer treatment, in combination with olaparib (Lynparza^®^)) [58]. In the following, we will present the latest outcomes regarding new quinazoline derivatives as VEGFR-2 inhibitors.

**Scheme 19:**
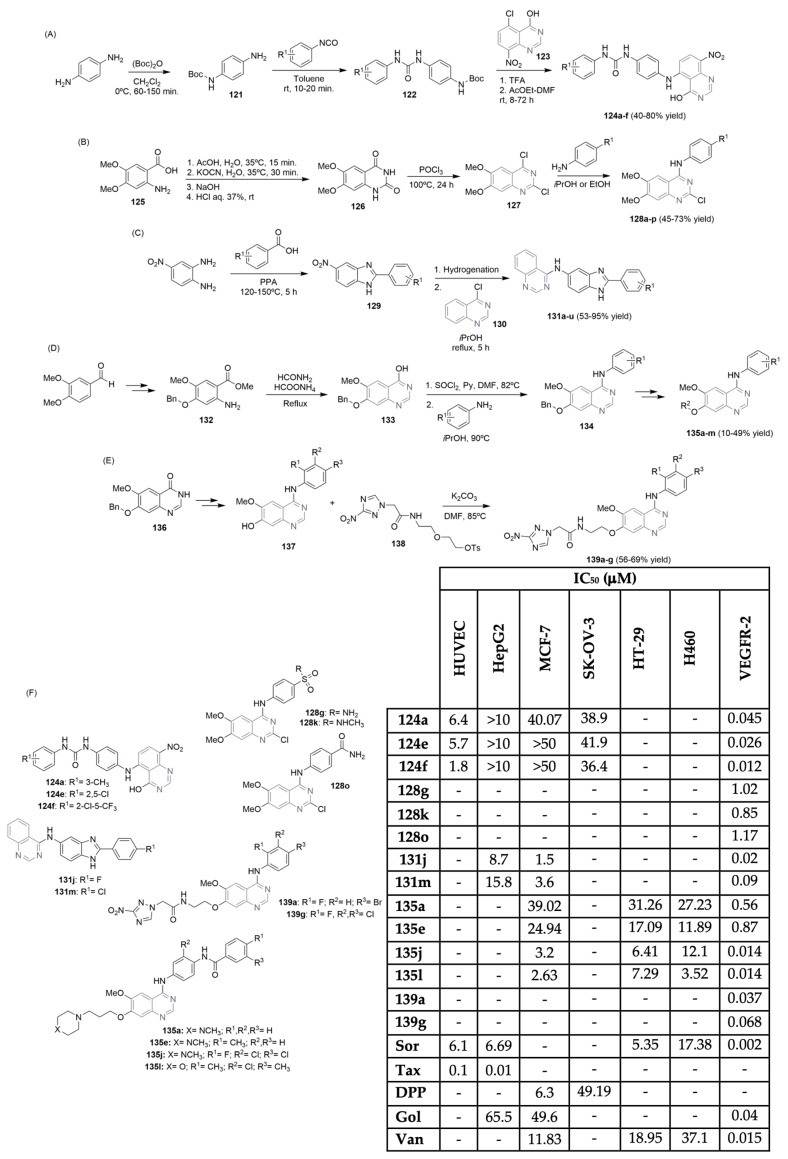
Synthesis of new 5-anilinoquinazoline-8-nitro (**A**), 2-chloro-4-anilinoquinazoline (**B**), quinazolin-4-amine (**C**), 4-anilinoquinazoline (**D**,**E**) derivatives and bioprofile (**F**). (PPA: polyphosphoric acid; HUVEC: human umbilical vein endothelial cells; Hep-G2: liver cancer cells; MCF-7: breast cancer cells; SK-OV-3: ovarian cancer cells; HT-29: colorectal cancer cells; H460: lung cancer cells; Sor: sorafenib; Tax: taxol; DPP: cisplatin; Gol: golvatinib; Van: vandetanib).

Jin, Lin, and co-workers described the synthesis of a library of new 5-anilinoquinazoline-8-nitro derivatives with several C-5 aryl urea substituents and tested their profiles as VEGFR-2 inhibitors (Scheme 19A,F) [59]. The single *N*-Boc protected diamines **121** were easily obtained from the aromatic diamine and Boc anhydride. After reacting with the corresponding isocyanates, the *N*,*N*′-disubstituted urea intermediates **122** were obtained in high yields, with short reaction times (Scheme 19A). After a two-step procedure, which included *N*-Boc deprotection and nucleophilic substitution with 5-chloro-quinazoline-8-nitro **123**, the corresponding 5-anilinoquinazoline-8-nitro analogs **124a**–**f** were obtained in moderate to good yields (Scheme 19A). The antiproliferative activity against four human cell lines, along with enzymatic inhibition of VEGFR-2, was evaluated for the library of 5-anilinoquinazoline-8-nitro derivatives **124a**–**f** (Scheme 19F). Compounds **124a**, **124e**, and **124f** were found to inhibit human umbilical vein endothelial cell (HUVEC) proliferation, but no antiproliferative activity against hepatocellular carcinoma cells (HepG2) was found. The same was observed regarding breast cancer (MCF-7) and ovarian cancer (SK-OV-3) cells, compared to cisplatin (DPP), the positive control (Scheme 19F). The 2,5-dichloro and 2-chloro-5-tri-fluoromethyl derivatives **124e** and **124f**, respectively, were the most potent inhibitors of the VEGFR-2 kinase, but were still less potent than that of sorafenib (Figure 1), the positive control (IC_50_ value of 0.002 μM compared to 0.026 μM for **124e** and 0.012 μM for **124f**; see Scheme 19F). Barreiro and co-workers reported on the synthesis and bioprofile of new 2-chloro-4-anilinoquinazoline derivatives and evaluated their VEGFR-2 inhibitory effects (Scheme 19B,F) [60]; 2,4-dichloro-quinazoline **127** was used as a key intermediate to obtain the corresponding 4-anilinoquinazoline derivatives **128a**–**p** in moderate to good yields (Scheme 19B). Anthranilic acid derivative **125** was used as a starting precursor to access intermediate **127**. The VEGFR-2 inhibitory effect of the quinazoline library **128** was evaluated, revealing compounds **128g**, **128k**, and **128o** as the most potent ones, with IC_50_ values of 1.02, 0.85, and 1.17 μM, respectively (Scheme 19F). Molecular docking studies demonstrated the importance of a hydrogen bond donor at the *para* position of the aniline unit for interaction with the VEGFR-2 binding site, promoting the increase in potency. Quinazolin-4-amines bearing benzimidazole moieties were synthesized and evaluated as VEGFR-2 inhibitors by Shi, Xu, and co-workers (Scheme 19C,F) [61]. Commercially available substituted benzoic acids and 4-nitro-*o*-phenylenediamine were the starting precursors to access 5-nitro-benzimidazole intermediates **129**, which—after hydrogenation and condensation with 4-chloroquinazoline **130**—yielded the desired *N*-(2-phenyl-1*H*-benzo[*d*]imida-zol-5-yl)quinazolin-4-amine derivatives **131a**–**u** in moderate to excellent yields (Scheme 19C). Among these compounds bearing quinazoline and benzimidazole units, compounds **131j** and **131m** exhibited the most potent inhibitory activities against VEGFR-2, with IC_50_ values of 0.02 and 0.09 μM, respectively. Golvatinib was used as the positive control, with an IC_50_ value of 0.04 μM (Scheme 19F). Evaluation regarding antiproliferative activity against human breast (MCF-7) and liver (Hep-G2) cancer cells highlighted compound **131j** as the most promising one, with IC_50_ values of 1.5 μM compared to MCF-7 and 8.7 μM compared to Hep-G2, better than golvatinib (Scheme 19F). Sun et al. described the design, synthesis, and VEGFR-2 evaluation of 4-anilinoquinazoline-acylamino and -urea derivatives (Scheme 19D) [62,63]. The key intermediate, 4-hydroxy-6-methoxy-7-benzyloxyquinazoline **133**, was obtained in several reaction steps, starting with 3-methoxy-4-benzyloxybenzaldehyde. Cyclized compound **133** reacted with thionyl chloride to obtain the corresponding chloroquinazoline; after nucleophilic substitution, the reaction with anilines yielded the corresponding intermediates **134**. In the subsequent two-step reaction approach (benzyl-removing and alkylation reaction), the desired 4-anilinoquinazoline-acylamino or -urea derivatives **135a**–**m** were obtained in poor to moderate yields (Scheme 19D). Antiproliferative activities of the synthesized libraries of **135** were evaluated toward human colorectal adenocarcinoma cells (HT-29), human breast cancer cells (MCF-7), and human lung cancer cells (H460). Compounds **135a** and **135e** demonstrated moderate antiproliferative effects against HT-29, MCF-7, and H460 cells, but the most potent inhibitory activity against VEGFR-2 (IC_50_ values of 0.56 and 0.87 μM, respectively), using sorafenib (Figure 1) and vandetanib (Figure 2) as positive controls (IC_50_ values of 0.01 μM and 0.015 μM) (Scheme 19F). Compounds **135j** and **135l** exhibited better activities against the three cell lines (IC_50_ values <12 μM), with chlorine in the *ortho*-position of the urea group. Regarding VEGFR-2 activity, an IC_50_ value of 0.014 μM for compounds **135j** and **135l** was obtained, very close to vandetanib (Figure 2) (IC_50_ of 0.015 μM), the positive control (Scheme 19F). Qin, Liu, Li, and co-workers described the synthesis and biological evaluation of novel 4-anilinoquinazolines with a 3-nitro-1,2,4-triazole unit in the side chain (Scheme 19E,F) [64]. Starting from 7-benzyloxy-6-methoxy-3*H*-quinazolin-4-one **136**, and after a two-step reaction approach, the intermediates **137** were easily obtained and in moderate yields. The previously prepared intermediate **138** (from 3-nitro-1*H*-1,2,4-triazole starting material) reacted with intermediate derivatives **137**, yielding the desired target compounds **139a**–**g** in moderate yields (Scheme 19E). The abilities of targeted compounds **139** to inhibit VEGFR-2 were evaluated via the kinase inhibitory assay, with vandetanib (Figure 2) as the positive control (Scheme 19F). Compounds **139a** and **139g** exhibited promising VEGFR-2 inhibitory activities, with IC_50_ values of 0.037 and 0.068 μM, respectively. Moreover, compound **139a** showed similar activity to vandetanib, with an IC_50_ of 0.033 μM (Scheme 19F). Molecular docking studies reinforced the great binding affinity of compound **139a** to the VEGFR-2 enzyme.

**Scheme 20:**
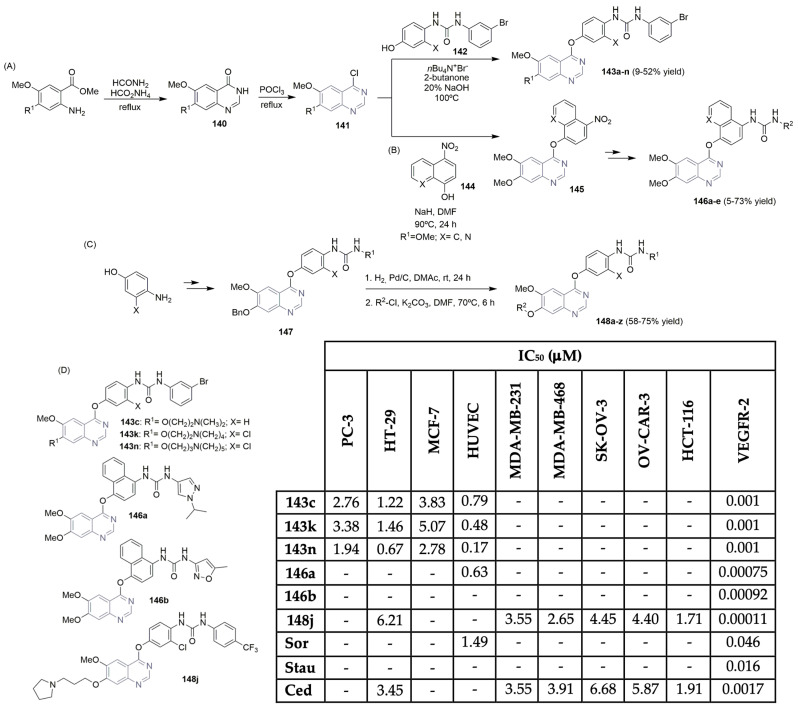
Synthesis of new 6-methoxy-4-aryloxiquinazoline ureas (**A**–**C**) and bioprofile (**D**). (PC-3: prostate cancer cells; HT-29: colorectal cancer cells; MCF-7, MDA-MB-231 and MDA-MB-468: breast cancer cells; HUVEC: human umbilical vein endothelial cells; SK-OV-3 and OV-CAR-3: ovarian cancer cells; HCT-116: colon cancer cells; Sor: sorafenib; Stau: staurosporine; Ced: cediranib).

New 4-aryloxy-6,7-dimethoxyquinazolines were synthesized and evaluated for their VEGFR-2 kinase activity and antiproliferative effects in several cancer cell lines (Scheme 20A,D) [65]. Cyclization of the 2-aminobenzoester with formamide in the presence of sodium formate yielded the cyclized intermediate **140**, in moderate yields. The key 4-chloroquinazoline intermediates **141** were easily obtained from **140** with POCl_3_. The desired urea-derived products **143** were obtained from the reaction between chloride derivatives **141** and the appropriate phenol-urea derivatives **142**, in the presence of tetra-*N*-butylammonium bromide in 2-butanone and a 20% solution of sodium hydroxide mixture, in low yields (Scheme 20A). Biological evaluation of the synthesized 7-aminoalkoxy-4-aryloxy-quinazoline urea derivatives **143** concerning their inhibitory activity on VEGFR-2 and antiproliferative activity on prostate (PC-3), colorectal (HT-29), and breast (MCF-7) cancer cell lines led to the identification of compounds **143c**, **143k** and **143n** as the most promising ones (Scheme 20D). A basic side chain substituted on the 7-position of the quinazoline scaffold, like diethylamino-alkoxy **143c**, piperidino-alkoxy **143n**, or pyrrolidino-alkoxy **143k**, led to potent VEGFR-2 inhibitors, revealing IC_50_ values on a nanomolar range (Scheme 20D). Also, significant antiproliferative activities were noted for these compounds on PC-3, HT-29, and MCF-7 cancer cell lines (IC_50_ values < 5 μM). Unfortunately, the same potency was verified for normal cells, HUVECs (Scheme 20D). Several years later, Lu, Jiao, and co-workers described the synthesis and VEGFR-2 activity of a new library of 4-aryloxy-6,7-dimethoxyquinazolines, applying computational and experimental studies (Scheme 20B,D) [66]. The synthesis of the target compounds started with the nucleophilic substitution reaction of the phenol intermediate **144** and 4-chloro-6,7-dimethoxyquinazoline **141**, yielding the key intermediate **145**. Reduction of **145** via iron powder provided the corresponding amino compounds, which reacted with different anilines to obtain the target urea derivatives **146a**–**e** in low yields (Scheme 20B). The small library of relatively high predicted binding activities to VEGFR-2 was evaluated for *in vitro* activity (Scheme 20D). Most of the target compounds **146** displayed nanomolar IC_50_ values against VEGFR-2; compounds **146a** and **146b** were the best ones (with IC_50_ values of 0.00075 and 0.00092 μM, respectively), but compound **146a** was also potent against the normal cell line, HUVEC (Scheme 20D). Sorafenib (Figure 1) and staurosporine were used as positive controls. Recently, Wang, Wu, Zhang, Wei, and Yang reported on the synthesis of a new series of substituted 4-anilinoquinazolines as potent VEGFR-2 inhibitors (Scheme 20C,D) [67]. Using an amino-phenol building block, several urea derivatives were prepared using the key intermediate **147**; after hydrogenation to remove the benzyl-protecting group, followed by etherification with several aliphatic chains, the target derivatives **148a**–**z** were obtained in moderate yields (Scheme 20C). Once again, a pyrrolidino-alkoxy basic side chain substituted on the 7-position of the quinazoline scaffold increases the potency of the compound regarding VEGFR-2 inhibition. So, compound **148j** was selected as a potent (IC_50_ value of 0.00011 μM) and selective VEGFR-2 inhibitor (Scheme 20D). Also, a good antiproliferative profile was obtained for compound **148j** in several cancer cell lines, like breast (MDA-MB-231 and MDA-MB-468), ovarian (SK-OV-3 and OV-CAR-3), and colorectal (HCT-116 and HT-29) (Scheme 20D), compared to cediranib (Figure 2), the positive control used.

**Scheme 21:**
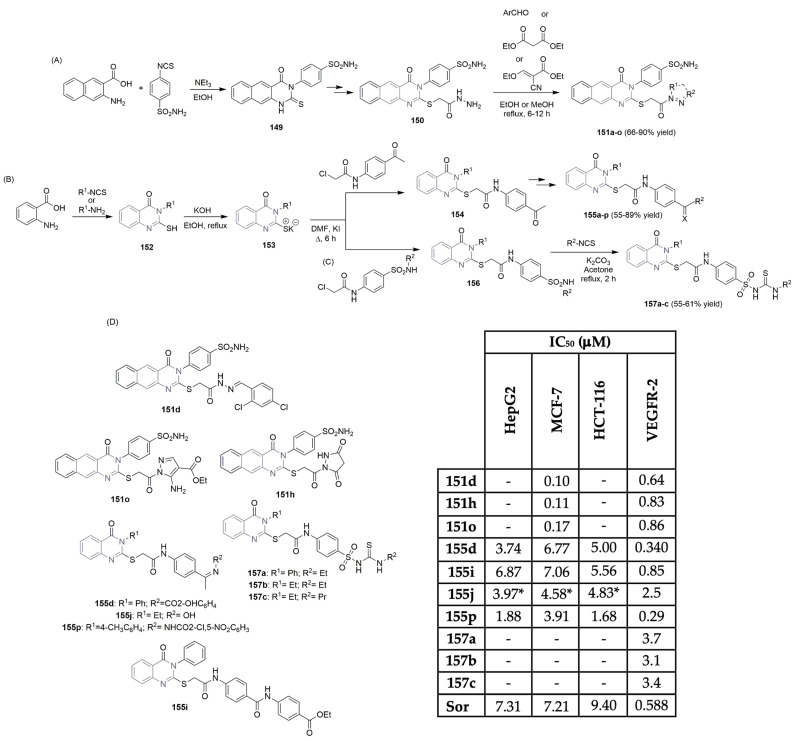
Synthesis of new benzo[*g*]quinazolin bearing benzenesulfonamide unit (**A**) and quinazolin-4(3*H*)-one derivatives (**B**,**C**) and bioprofile (**D**). (Hep-G2: liver cancer cells; MCF-7: breast cancer cells; HCT-116: colon cancer cells; Sor: sorafenib; * values in μg/mL).

Ghorab and co-workers described the synthesis of new benzo[*g*]quinazoline derivatives targeting VEGFR-2 (Scheme 21A,D) [8]. Starting from the reaction of 3-amino-2-naphthoic acid with 4-isothiocyanatobenzenesulfonamide, the key intermediate 4-(2-mercapto-4-oxo-benzo[*g*]quinazolin-3(4*H*)-yl) benzenesulfonamide **149** was obtained in good yield. A nucleophilic substitution reaction, followed by a reaction with hydrazine hydrate in ethanol, gave access to the key intermediate **150** (Scheme 21A). The reaction of **150** with several reagents, like benzaldehyde derivatives, diethyl malonate, or ethyl 2-cyano-3-ethoxyacrylate, yielded the target benzo[*g*]quinazoline-benzenesulfonamide derivatives **151a**–**o** in moderate to good yields (Scheme 21A). After *in situ* screening of the library for cytotoxic activity against breast cancer cell line MCF-7 and evaluation of VEGFR-2 enzyme inhibition, the authors concluded that compounds **151d**, **151h**, and **151o** were the most potent (Scheme 21D). IC_50_ values of >0.9 μM were obtained in the VEGFR-2 inhibition assay, lower than that of vandetanib (Figure 2), the positive control. Compounds **151d**, **151h**, and **151o** also proved to be the most cytotoxic regarding the MCF-7 cell line, with IC_50_ values ranging from 0.10 to 0.17 μM (Scheme 21D). Eissa et al., who are very active in the design and discovery of new scaffolds as VEGFR-2 inhibitors, described the importance and potency of new quinazolin-4(3*H*)-one derivatives as VEGFR-2 inhibitors (Scheme 21B) [68,69,70]. Based on previously reported hit compounds, novel libraries of quinazoline-based derivatives were successfully synthesized, starting with anthranilic acid and aniline or the isothiocyanate derivative. The purpose was to increase the binding affinity of the designed compounds to the receptor’s active site. Moreover, 2-mercapto-3-phenyl-quinazolin-4(3*H*)-one **152** and the corresponding potassium salt **153** were the key building blocks used to access the target compounds. Various intermediates like *N*-(4-acetylphenyl)-2-chloroacetamide, hydrazine derivatives, etc., were used to access compounds **155a**–**p** in moderate to good yields (Scheme 21B). The new libraries were evaluated for their cytotoxic activity in liver, colon, and breast cancer cell lines (Hep-G2, HCT-116, and MCF-7, respectively), as well as for their inhibitory potency against the VEGFR-2 kinase (Scheme 21D). The most potent compounds were **155d**, **155i**, **155j**, and **155p** with IC_50_ values for the VEGFR-2 inhibitory assay in the range of 0.29 to 2.5 μM. The bioactivity against Hep-G2, HCT-116, and MCF-7 cell lines showed very good cytotoxicity for compound **155p**, with IC_50_ values of 1.88, 1.68, and 3.91 μM, respectively (Scheme 21D). Sorafenib (Figure 1) was used as the positive control. The same group (Eissa, Mahdy, et al.) reported on a new library of quinazolin-4(3*H*)-one derivatives featuring sulfonamide units and evaluated their activity regarding VEGFR-2 (Scheme 21C,D) [71]. A similar synthetic pathway was implemented in the synthesis of derivatives **157a**–**c**, obtained with moderate yield. Compounds **157a**–**c** revealed good activity as VEGFR-2 inhibitors (IC_50_ values ranging from 3.1 to 3.7 μM); however, they were less potent than the analogous **155** (Scheme 21D).

**Scheme 22:**
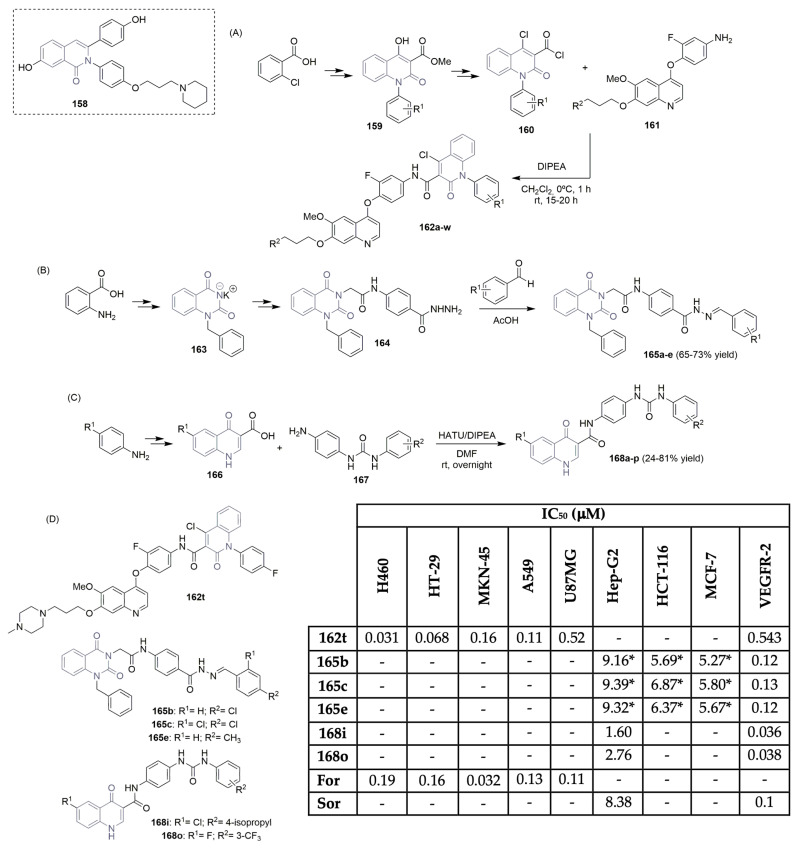
Synthesis of new 2,3-diaryl isoquinolinone (**A**), 1-benzylquinazoline-2,4(1*H*,3*H*)-dione (**B**) and quinolone-3-carboxamide (**C**) derivatives and bioprofile (**D**). (H460 and A549: lung cancer cells; HT-29 and HCT-116: colorectal cancer cells; MKN-45: gastric cancer cells; U87MG: glioblastoma cancer cells; Hep-G2: liver cancer cells; MCF-7: breast cancer cells; For: foretinib; Sor: sorafenib; * GI_50_ values; HATU: hexafluorophosphate azabenzotriazole tetramethyl uronium).

Xiang et al. reported interesting work regarding the importance of isoquinolinone/isoquinolone units targeting VEGFR-2 anticancer agents [72,73,74]. The 2,3-diaryl isoquinolinone derivative **158** (Scheme 22A) was used as the hit compound since it exhibited significant VEGFR-2 activity (100.26% inhibition at 0.1 mg/mL) and potent cytotoxicity in breast cancer cell MCF-7 (IC_50_ value of 2.73 μM) [72]. A new library of 6-aryl-indenoisoquinolone derivatives was synthesized and evaluated regarding VEGFR-2 inhibitory potency (Scheme 22A,D). The key intermediate **159** was obtained from commercially available 2-chlorobenzoic acid in a three-step reaction approach, consisting of the Ullmann reaction, acylation, and condensation. Hydrolysis and acyl chlorination reactions were used to convert **159** to the corresponding acyl chlorides **160**, which reacted with the key intermediates of 6,7-disubstituted-4-phenoxyquinolines **161**, yielding the target quinoline derivatives, bearing a 2-oxo-4-chloro-1,2-dihydroquinoline-3-carboxamide moiety, labeled as **162a**–**w** (Scheme 22A). The library was *in vitro*-tested in five human cell lines, lung (H460 and A549), colorectal (HT-29), gastric (MKN-45), and glioblastoma (U87MG) (Scheme 22D). Compound **162t** showed the strongest cytotoxicity activities, with IC_50_ values of 0.031, 0.11, 0.068, 0.16, and 0.11 μM, respectively. Regarding H460, HT-29, and U87MG, compound **162t** demonstrated better potency than foretinib, the positive control. However, compound **162t** revealed weak potency against VEGFR-2 (IC_50_ value of 0.543 μM (Scheme 22D). El-Adl et al. described the synthesis of novel 1-benzylquinazoline-2,4(1*H*,3*H*)-diones and evaluated their anticancer activity (Scheme 22B,D) [75]. Anthranilic acid was used as the starting material, which underwent a two-step reaction sequence to access the potassium salt of 1-benzylquinazoline-2,4(1*H*,3*H*)-dione **163**. The consequent alkylation and reaction with hydrazine hydrate provided the corresponding key intermediate hydrazine derivative **164**. Condensation of **164** with benzaldehyde derivatives yielded the target Schiff bases **165a**–**e**, in moderate yields (Scheme 22B). Compounds **165b**, **165c**, and **165e** were found to be the most potent against liver (Hep-G2), colorectal (HCT-116), and breast (MCF-7) cancer cell lines, with GI_50_ values in the range of 5.27 to 9.39 μM. Regarding enzymatic inhibitory activity against VEGFR-2, compounds **165b**, **165c**, and **165e** demonstrated high potency, with IC_50_ values of 0.12, 0.13 and 0.12 μM, very close to sorafenib (Figure 1) (IC_50_ value of 0.1 μM) (Scheme 22D). Molecular docking studies supported the binding patterns of the most potent compounds toward the active VEGFR-2 site. Recently, Arafa, Abou-Seri, and co-workers described the synthesis and VEGFR-2 inhibition profile of new quinolone-3-carboxamide hybrids (Scheme 22C,D) [76]. The synthesis of the quinolone ring was accomplished via the Gould–Jacobs reaction, using substituted anilines as the starting material. Key intermediate 4-oxo-1,4-dihydroquinoline-3-carboxylic acid derivatives **166** were prepared via a three-step reaction sequence. The reaction of 1-(4-aminophenyl)-3-arylurea derivatives **167** and the quinolinone-3-carboxylic acid derivatives **166** yielded the target 6-substituted-4-quinolone-3-carboxamide derivatives **168a**–**p**, in low to good yields, using the coupling agents HATU (hexafluorophosphate azabenzotriazole tetramethyl uronium) and DIPEA (*N*,*N*-diisopropylethylamine) as bases (Scheme 22C). Compounds **168i** and **168o** were the most potent regarding VEGFR-2 inhibition activity, with IC_50_ values of 0.036 and 0.038 μM, close to sorafenib (Figure 1), the positive control, which had an IC_50_ value of 0.045 μM (Scheme 22D). Additionally, compounds **168i** and **168o** demonstrated good cytotoxicity against the liver cell line Hep-G2, with IC_50_ values lower than sorafenib, the positive control (compare IC_50_ values of 1.60 and 2.76 μM for compounds **168i** and **168o**, respectively, and IC_50_ value of 8.38 μM for sorafenib) (Scheme 22D).

**Scheme 23:**
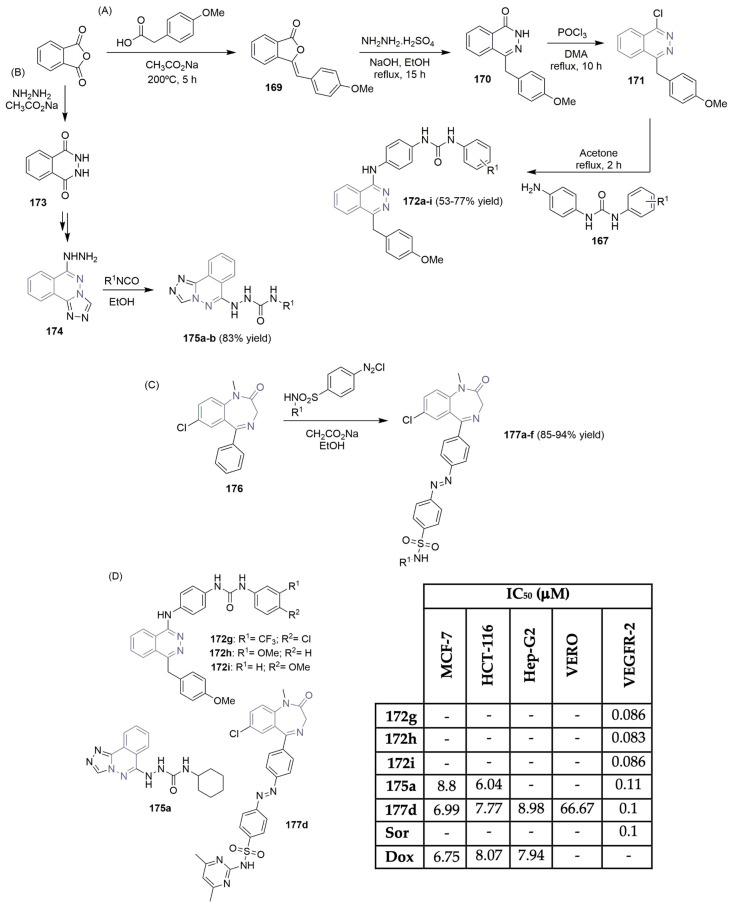
Synthesis of new phthalazine (**A**,**B**), and diazepam-sulfonamide (**C**) derivatives and bioprofile (**D**). (MCF-7: breast cancer cells; HCT-116: colorectal cancer cells; Hep-G2: liver cancer cells; VERO: normal kidney cells; Sor: sorafenib; Dox: doxorubicin).

Eldegna, Abou-Seri, and co-workers described the design, synthesis, and VEGFR-2 activity of 1-substituted-4-(4-methoxybenzyl) phthalazine derivatives (Scheme 23A,D) [77]. A library of anilinophthalazine hybrids **172a**–**i** was synthesized using the commercially available phthalic anhydride and 4-methoxyphenyl acetic acid to give the first precursor, 3-(4-methoxybenzylidene)isobenzofuran-1(3*H*)-one **169**. Treatment with hydrazine hydrate gave access to intermediate **170**; after chlorination with POCl_3_, this yielded the key intermediate 1-chloro-4-(4-methoxybenzyl)phthalazine **171**. The target anilinophthalazine hybrids **172a**–**i** were prepared by refluxing **171** with the urea intermediate **167** in acetone (Scheme 23A). The VEGFR-2 inhibitory assay revealed that the potency of compounds **172g**, **172h**, and **172i**, with IC_50_ values of 0.086, 0.083, and 0.086 μM, were very close to the positive control sorafenib (IC_50_ of 0.1 μM) (Scheme 23D). The substituted phenyl moiety through a urea linker increased the hydrophobic interaction with the hydrophobic back pocket of the active VEGFR-2 site. These results were confirmed via molecular modeling studies. El-Adl and co-workers also described the synthesis and anticancer activities of new phthalazine derivatives (Scheme 23B,D) [78]. Also, using the phthalic anhydride as starting material, the 2,3-dihydrophthalazine-1,4-dione intermediate **173** was easily obtained using hydrazine hydrate. A four-step reaction sequence led to the key intermediate 6-hydrazinyl-[1,2,4]triazolo[3,4-*a*]phthalazine **174**, which was refluxed with the appropriate isocyanate to yield the target carboxamide derivatives **175a**–**b**, in good yield (Scheme 23B). The phthalazine derivatives were tested for their antiproliferative activities in two human cancer cell lines, colon (HCT-116) and breast (MCF-7). Compound **175a** revealed closed IC_50_ values that were comparative to sorafenib (Figure 1), the positive control (with IC_50_ values of 6.04 and 8.8 μM for 175a, and 5.47 and 7.26 μM for sorafenib, for HCT-116 and MCF-7, respectively) (Scheme 23D). Also, similar VEGFR-2 inhibitory activity was found for compound **175a**, a little bit higher than for the phthalazine derivatives **172** (Scheme 23D). El-Sattar and co-workers studied VEGFR-2’s inhibitory activity and antiproliferative activity on diazepam-bearing sulfonamide unit hybrids (Scheme 23C,D) [79]. The synthesis of the target compounds **177a**–**f** involved the addition of the key 4-aminobenzenesulfonamide diazonium salt to diazepam **176** in EtOH with sodium acetate (Scheme 23C). Evaluation of the cytotoxicity of the library against liver (Hep-G2), colon (HCT-116), and breast (MCF-7) cancer cells highlighted compound **177d** as the most potent. IC_50_ values very close to doxorubicin (the positive control) were obtained for **177d** (Scheme 23D). Regarding the VEGFR-2 inhibitory assay, the same compound, **177d**, proved to be very potent, with an IC_50_ value of 0.1 μM, which was the same for sorafenib (Figure 1), the positive control (Scheme 23D).

**Scheme 24:**
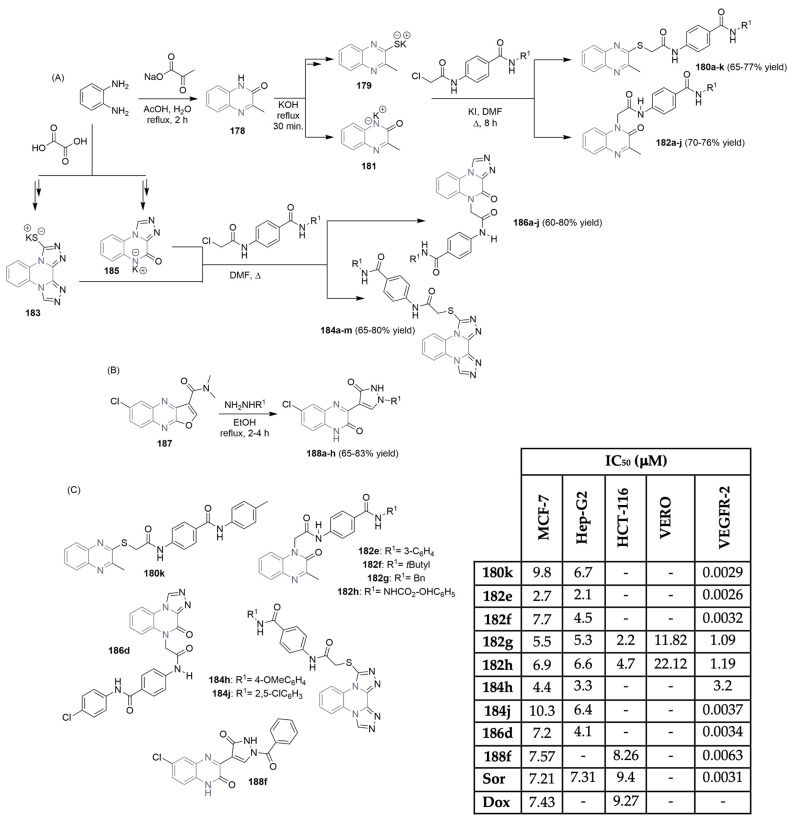
Synthesis of new quinoxaline derivatives (**A**,**B**) and bioprofile (**C**). (MCF-7: breast cancer cells; Hep-G2: liver cancer cells; HCT-116: colorectal cancer cells; VERO: normal kidney cells; Sor: sorafenib; Dox: doxorubicin).

Alanazi, Eissa, et al. presented new libraries of quinoxaline hybrids and evaluated their potential as anticancer agents (Scheme 24A,C) [80,81,82,83,84,85]. In the first synthetic approach, *ortho*-phenylenediamine was refluxed with sodium pyruvate, yielding 3-methylquinoxalin-2(1*H*)-one **178**. The subsequent two-step reaction approach gave the corresponding potassium salt **179**, which reacted with the key chlorine intermediates, yielding the target 3-methylquinoxalines **180a**–**k** (Scheme 24A). The potassium salt of intermediate **178** was easily obtained by refluxing **178** with KOH, yielding the intermediate salt **181**. The same procedure, using chlorine intermediate in DMF and a catalytic amount of KI, gave access to the target derivatives **182a**–**j**, achieving good yields (Scheme 24A). Similar reaction procedures were used to access the bis([1,2,4]triazolo)[4,3-*a*:3′,4′-*c*]quinoxaline derivatives **184** and **186** (Scheme 24A) [83,84,85]. The same *ortho*-phenylenediamine was used as the starting material, with oxalic acid accessing the key potassium salt intermediates **183** and **185**. Cytotoxicity and VEGFR-2 inhibitory activities were evaluated for the families of quinoxazoline derivatives (Scheme 24C). Moreover, the 3-methylquinoxalin-2(1*H*)-one derivative **182e** and 3-methylquinoxaline-2-thiol derivative **180k** were the most promising compounds as VEGFR-2 inhibitors (IC_50_ values of 0.0026 and 0.0029 μM). The bis([1,2,4]triazolo)[4,3-*a*:3′,4′-*c*]quinoxaline derivative **184h** was less potent in the VEGFR-2 assay (Scheme 24C). Some quinoxaline derivatives were also evaluated toward cytotoxicity against breast (MCF-7), liver (Hep-G2), and colorectal (HCT-116) human cancer cells. The 3-methylquinoxalin-2(1*H*)-one derivative **182e** was the most cytotoxic compound against breast and liver cancer cell lines, with IC_50_ values of 2.7 and 2.1 μM, respectively. Compound **182g** was the most potent regarding colorectal cancer cells, with an IC_50_ of 2.2 μM. Sorafenib (Figure 1) and doxorubicin were used as positive controls in this assay. Very recently, Ismail and co-workers described the synthesis and VEGFR-2 inhibitory activities of a new library of quinoxaline-2-one derivatives (Scheme 24B,C) [86]. The key intermediate quinoxazoline **187** reacted with several hydrazine derivatives, yielding the quinoxazoline-based pyrazolones **188a**–**h**, in moderate to good yields (Scheme 24B). After exploring the VEGFR-2 inhibitory potency of the library, compound **188f** was the most potent inhibitor of VEGFR-2, with an IC_50_ value of 0.0063 μM; see Scheme 24C. Also, compound **188f** demonstrated good cytotoxicity against breast (MCF-7) and colorectal (HCT-116) human cancer cell lines (with IC_50_ in the range of 7.5 to 8.5 μM), using doxorubicin as the positive control.

### 2.3. Coumarin-Hybrid Scaffolds

**Scheme 25:**
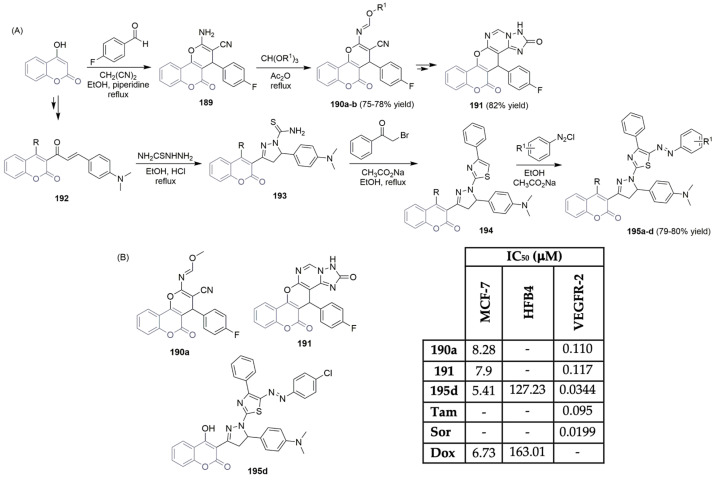
Synthesis of new coumarin derivatives (**A**) and bioprofile (**B**). (MCF-7: breast cancer cells; HFB4: normal human melanocyte cell line; Tam: tamoxifen; Sor: sorafenib; Dox: doxorubicin).

The coumarin scaffold is considered a privileged unit in drug design and discovery because it is found in many naturally occurring substances. Its synthetic flexibility, physicochemical features, and ease of modification make this peculiar unit a good candidate for developing a large variety of functionalized coumarin hybrids [87]. In the context of this review, several coumarin derivatives demonstrated powerful cytotoxicity properties, which resulted in efficient anticancer agents [88]. Batran and co-workers described the synthesis and anticancer effects of new coumarin hybrids, highlighting VEGFR-2 activity (Scheme 25) [89,90]. Both the synthetic approach for accessing the target pentacyclic triazolopyrimidine coumarin hybrid **191** and the thiazolyl–pyrazolyl coumarin derivatives **195a**–**d** began with the use of 4-hydroxycoumarin (Scheme 25A). On the first synthetic path, a three-component one-pot reaction of 4-hydroxycoumarin, 4-fluorobenzaldehyde, and malononitrile in refluxing EtOH yielded the key intermediate 2-amino-3-cyanopyranocoumarin **189**, which reacted with trimethyl or ethyl orthoformate to yield the corresponding tricyclic derivative **190a**–**b**. The target pentacyclic triazolopyrimidine coumarin hybrid **191** was obtained in a two-step reaction sequence, with good yield (Scheme 25A). To obtain the target thiazolyl–pyrazolyl coumarin derivatives **195**, 4-hydroxycoumarin was also the starting material. The 4-dimethylaminophenyl chalcone intermediate **192** was synthesized through a multi-step reaction sequence, starting from 4-hydroxycoumarin. Cyclization of **192** with thiosemicarbazide yielded the thiosemicarbazide intermediate **193**, in the presence of catalytic amounts of HCl. Condensation of **193** with several α-halo ketones through S-alkylation, and subsequent water or alcohol molecule elimination, led to the intermediates **194**. Coupling with the diazotized anilines yielded the target azo derivatives **195** (Scheme 25A). From all the coumarin hybrids tested for their cytotoxicity against breast cancer cells (MCF-7), compounds **190a**, **191**, and **195d** demonstrated to be the most potent ones (IC_50_ values of 8.28, 7.90, and 5.41 μM). Regarding VEGFR-2 inhibitory activity, the thiazolyl–pyrazolyl coumarin derivative **195d** was the most potent, with an IC_50_ value of 0.0344 μM. Sorafenib (Figure 1) was used as the positive control, with an IC_50_ of 0.0199 μM (Scheme 25B). No clear toxicity was observed toward normal cells, HFB4.

**Scheme 26:**
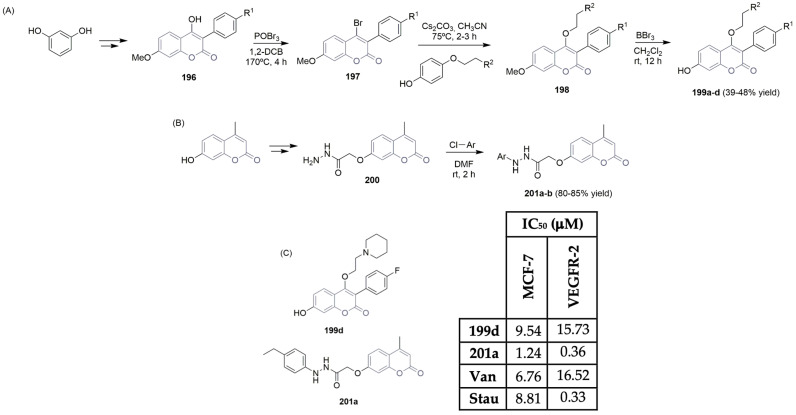
Synthesis of new coumarin derivatives (**A**,**B**); breast and VEGFR-2 evaluations of the most active compounds (**C**). (MCF-7: breast cancer cells; Van: vandetanib; Stau: staurosporine).

Xiang et al. described the synthesis of new 3-aryl-4-anilino/aryloxy-2*H*-chromen-2-one derivatives and evaluated them as VEGFR-2 inhibitors (Scheme 26A,C) [91]. Using resorcinol as the starting material, intermediate **196** was obtained in a multi-step reaction approach comprising Friedel–Craft and Mitsunobu reactions, among others. The 4-hydroxycoumarin derivative **196** was subjected to a bromination reaction with phosphorous oxybromide, giving the intermediate **197**. The coupling of **197** with several 4-[(2-aminoethoxy)]-substituted phenols yielded the key intermediate **198**, which—by demethylation with BBr_3_—yielded the target 4-*O*-linked 3-aryl-4-aryloxy-2*H*-chromen-2-one derivatives **199a**–**d**, in moderate yields (Scheme 26A). The evaluation regarding VEGFR-2 inhibitory activity revealed the potency of compound **199d** (IC_50_ of 15.73 μM), which was higher than that of the positive control vandetanib (Figure 2) (IC_50_ of 16.52 μM) (Scheme 26C). Compound **199d** had the best cytotoxic effect on the breast cancer cell line MCF-7, with an IC_50_ of 9.54 μM (Scheme 26C). Molecular docking revealed that compound **199d** could occupy the ATP region of the VEGFR-2 enzyme, forming a Π-Π stacking interaction and two hydrogen bonding interactions with key amino acid residues. Abdelhafez and co-workers also reported interesting work regarding new coumarin hybrids as anti-breast cancer agents (Scheme 26B,C) [92]. The coumarin hydrazide intermediate **200** was easily accessed from 7-hydroxy-4-methyl-2*H*-chromen-2-one via a multi-step reaction process. The reaction of hydrazide intermediate **200** with acid chlorides, like benzyl chloride and benzoyl chloride, among others, smoothly yielded the coumarin derivatives **201** (Scheme 26B). *In vitro* cytotoxic studies on the breast cancer cell line MCF-7 revealed promising cytotoxicity of compound **201a** (IC_50_ of 1.24 μM), superior activity compared to the positive control staurosporine (IC_50_ of 8.81 μM) (Scheme 26C). Remarkably, compound **201a** also exhibited significant inhibitory activity in the VEGFR-2 assay, with an IC_50_ of 0.36 μM, very close to the positive control staurosporine, which had an IC_50_ value of 0.33 μM; see Scheme 26C.

### 2.4. Isatin-Hybrid Scaffolds

The oxindole scaffold is considered a privileged structure in medicinal chemistry [93,94,95,96]. Isatin **202**, a synthetically versatile and cheap reagent, is often used in organic and medicinal chemistry as a starting material to attain libraries of compounds, as centrally explored by our group [97,98,99,100,101].

The oxindole moiety, which can be easily included in a larger chemical framework by using isatin, is present in sunitinib, a clinically approved VEGFR-2 inhibitor, and is often used by medicinal chemists to develop new potential drug candidates.

**Scheme 27:**
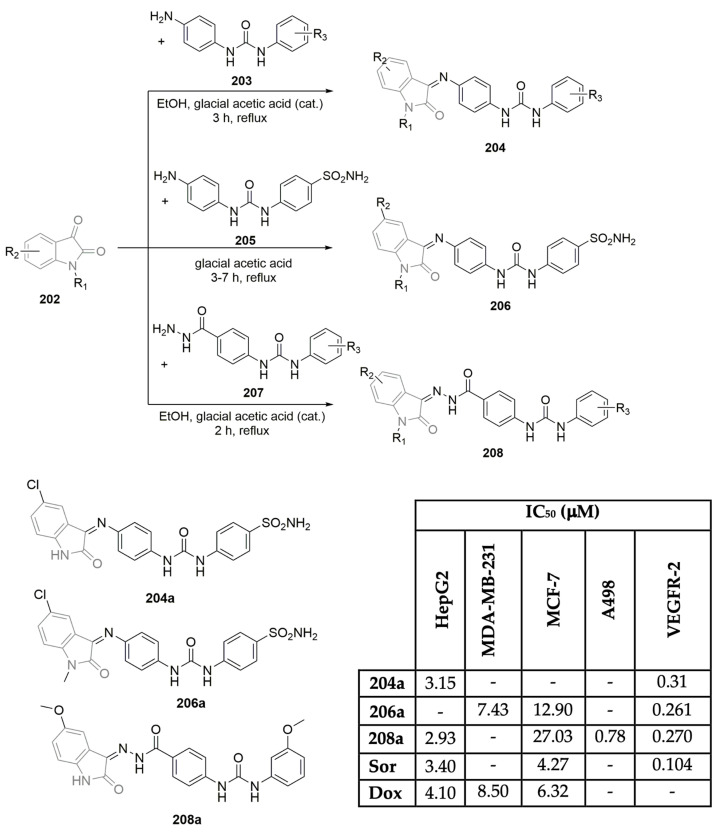
Synthesis of new oxindole-diphenyl urea hybrids with promising VEGFR-2 activity (Sor: sorafenib; Dox: doxorubicin).

Eldehna and co-workers reported multiple examples of isatin-based hybrid molecules with promising antiproliferative activity, targeting VEGFR-2. A library of 24 compounds was prepared by combining **202** (or 5-haloisatins) with 1-(4-aminophenyl)-3-phenylurea derivatives **203**, via the condensation of the primary amine group with the carbonyl group at position 3 of the **202** core. This straightforward synthetic procedure required catalytic glacial acetic acid in refluxing ethanol, achieving the desired products **204** in overall very good yields (up to 90%). Multiple compounds displayed promising antiproliferative activity against the hepatocellular carcinoma cell line (HepG2 cancer cells) at a lower micromolar range. The most promising hybrids were then evaluated for their inhibitory action toward VEGFR-2, with compound **204a** emerging as the strongest inhibitor. These results were further confirmed using molecular docking models, evaluating the ability of such a compound to bind to the active site of this kinase and comparing it to a standard inhibitor, sorafenib (Figure 1). The docking studies confirmed that compound **204a** possessed the highest score in the library, a result aligned with the observed *in vitro* evaluation [102]. With this knowledge, the structure of the most promising compound was picked up, namely, the 4-(3-(4-aminophenyl)ureido)benzenesulfonamide **205**, and researchers explored the potential of the *N*-substitution of the **202** moiety by replacing the hydrogen with alkyl and benzyl groups. The hybridization process unfolded using the same synthetic approach (library **206**). This method led to compound **206a**, which displayed good antiproliferative activity against MDA-MB-231 and MCF-7 cell lines (IC_50_ values of 7.43 and 12.90 μM, respectively) and potent VEGFR-2 inhibition at the nanomolar range (IC_50_ of 260.64 nM) [103]. Using the same synthetic approach but with different substitution patterns in the ureides **207** and oxindole moieties, a library was obtained, **208**, where compound **208a** displayed promising antiproliferative activity against HepG2 and A498 (with IC_50_ values of 2.93 and 0.78 μM, respectively). This compound demonstrated some selectivity, as it only displayed moderate activity against MCF-7 and no activity toward A549. The mechanism of action was proposed to be based on multi-kinase inhibition, as it was able to successfully inhibit VEGFR-2 (IC_50_ of 0.27 μM) as well as two other relevant targets (FGFR-1 and PDGFR-b at concentrations of 0.30 and 0.11 μM, respectively) (Scheme 27) [104].

**Scheme 28:**
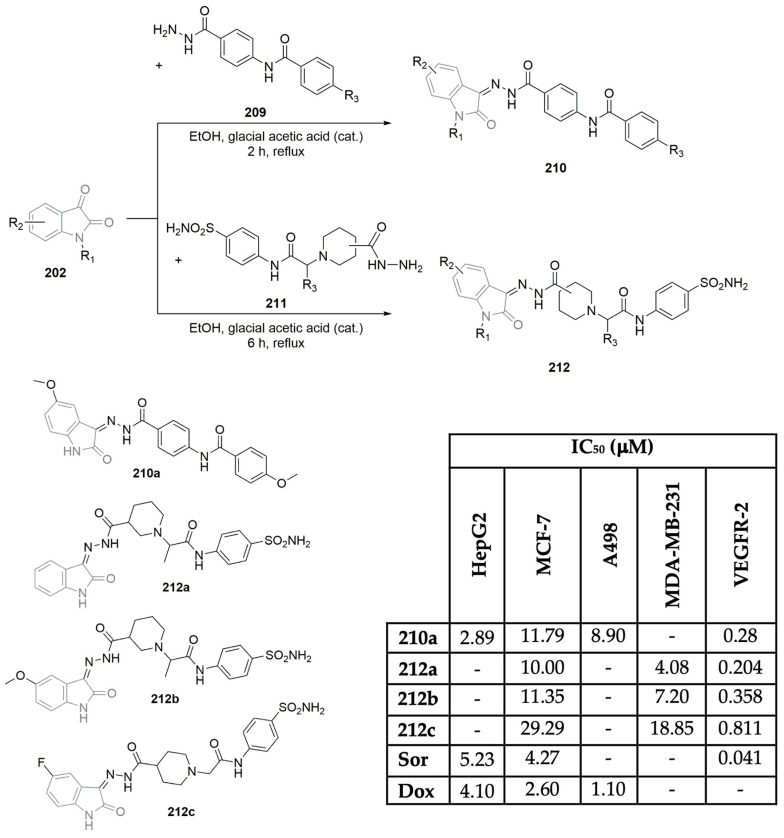
Synthesis of new oxindole-amide hybrids with promising VEGFR-2 activity (Sor: sorafenib; Dox: doxorubicin).

The same research group directed their attention to the use of the amide functional group instead of the urea functional group in the preparation of new oxindole derivatives. In one example, they coupled **202** (and aryl-substituted isatins) with *N*-(4-(hydrazinecarbonyl)phenyl)benzamides **209**, yielding a library of hybrids **210** (10 examples), in moderate to good yields (65–83%). Phenylbenzamide **209** was prepared through the coupling of 4-substituted benzoic acid and ethyl 4-aminobenzoate via EDCI, followed by the conversion of the ester group to the required hydrazide. The desired hybrids **210** were obtained using the aforementioned synthetic pathway, using catalytic glacial acetic acid in refluxing ethanol. The most promising compound, **210a**, displayed multi-kinase inhibitory activity, similar to what was reported for compound **208a**, including VEGFR-2 (IC_50_ of 0.28 μM). These findings were associated with antiproliferative activity, particularly against the HepG2 cell line (IC_50_ of 2.89 μM). The compound was also evaluated for its antiproliferative activity against other tumor cell lines (MCF-7, A549, and A498), with *in vitro* IC_50_ values ranging between 8.90 and 73.10 μM [104]. In another report, benzenesulfonamide hydrazides **211** were hybridized with isatins **202**, yielding a new library, **212** (20 examples), using the same synthetic strategy. The resulting compounds were evaluated for their carbonic anhydrase inhibition. The most promising compounds (**212a**, **212b**, and **212c**) were then assessed for their dual-inhibitory activity against VEGFR-2 and their antiproliferative effects, as depicted in Scheme 28 [105].

**Scheme 29:**
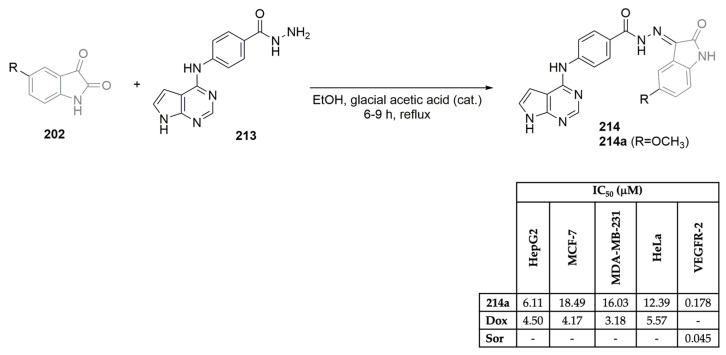
Synthesis of new 7-deazapurine–oxindole hybrids with promising VEGFR-2 activity (Sor: sorafenib; Dox: doxorubicin).

Alanazi and co-workers prepared a small library of isatin-based derivatives using the condensation reaction between isatins **202** and a 7-deazapurine-bearing hydrazide **213**. This synthesis, promoted by the presence of glacial acetic acid in refluxing ethanol, yielded five compounds, labeled **214**, with overall good yields (89–94%) (Scheme 29). From this library, **214a** emerged as the most promising compound, demonstrating strong antiproliferative activity in cancer cell lines. This activity was justified by its mechanism of action, which included the inhibition of multiple protein kinases, including VEGFR-2 [106].

**Scheme 30:**
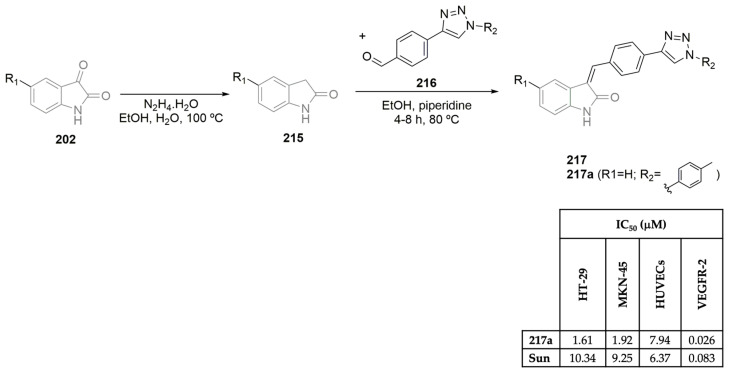
Synthesis of new triazole–oxindole hybrids with promising VEGFR-2 activity (Sun: sunitinib).

The synthesis of oxindole–triazole hybrids was reported by Wang and co-workers. The carbonyl group at position 3 of isatins **202** was reduced to the corresponding indolin-2-one derivatives **215** using hydrazine hydrate. In the next step, the Claisen–Schmidt condensation between the indolin-2-one derivatives and substituted 1,2,3-triazole aromatic aldehydes **216** yielded library **217** (28 examples), using piperidine as the catalytic base (Scheme 30). Compound **217a** proved to be the most promising from this library, as it displayed selective cytotoxic activity, with higher antiproliferative activity in tumor cell lines and lower toxicity toward human umbilical vein endothelial cells (HUVECs; non-tumor cell line) than sunitinib (Figure 1), which was used as the reference drug. *In vitro* assays and a molecular dynamic evaluation confirmed VEGFR-2 inhibition as the mechanism of action for this compound [107].

### 2.5. Urea–Thiourea-Hybrid Scaffolds

Urea and thiourea moieties are commonly used in medicinal chemistry and are present in a wide variety of bioactive molecules [108,109], including some examples already described in this work. They are present in VEGFR-2 inhibitors used in cancer therapeutics, such as sorafenib and regorafenib. In this section, we will explore a heterogeneous group of molecules that did not belong to previous sections; however, they all possess (thio)urea moieties in their chemical scaffolds.

**Scheme 31:**
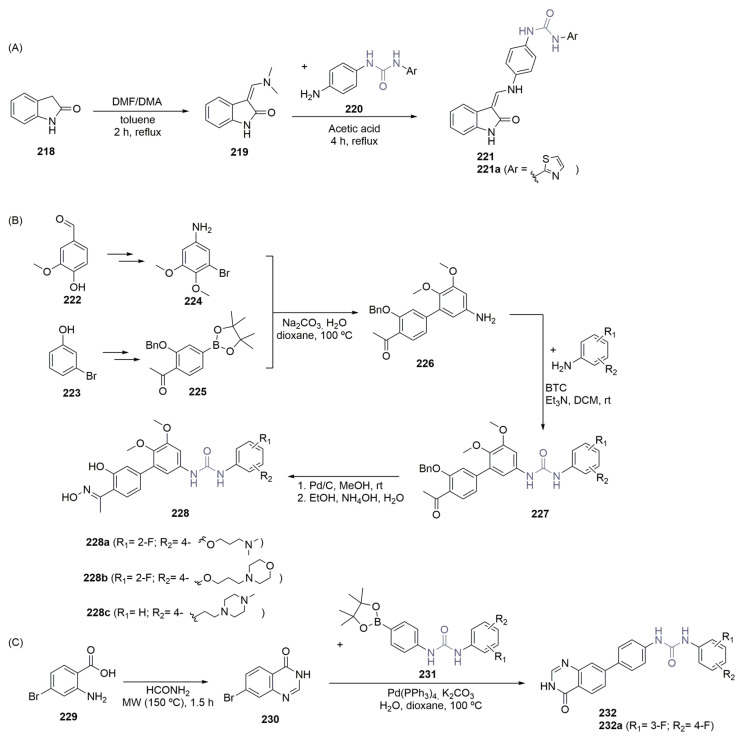
Synthesis of oxindole–urea hybrids (**A**) and diarylurea hybrids (**B**,**C**) with promising VEGFR-2 activity.

Ismail and co-workers explored the preparation of oxindole-based hybrids using 2-oxindole **218** instead of **202** as the starting material. Briefly, they converted 2-oxindole to an *N*-methylene intermediate **219**, which was then submitted to condensation with substituted anilines bearing the urea moiety **220**. The resulting library **221** (three examples) was obtained in moderate to good yields (68–84%) in a reaction promoted by glacial acetic acid. Compound **221a** emerged as the compound displaying higher cell growth inhibition using the NCI 60 cancer cell line panel (with mean growth inhibition of 61.83%). Further studies showed that **221a** presented high antiproliferative activity against tumor cell lines MCF-7, DU-145, and HCT-116 (in the low nanomolar ranges: 4.39, 1.06, and 0.34 nM, respectively), and its mechanism of action appeared to be multifactorial, as it could inhibit multiple kinases, including VEGFR-2 (73% inhibition at 10 μM). This mechanism was further validated by molecular docking studies (Scheme 31A) [110].

Recently, Zhang and co-workers dedicated efforts to urea-based compounds targeting VEGFR-2. In one example, they reported a library of biphenylurea derivatives bearing salicylaldoxime. The salicylaldoxime unit, capable of establishing an intramolecular hydrogen bond between the phenol group and the nitrogen atom of the aldoxime, was selected for its planar resemblance with bicyclic quinazoline. As previously described in this work, several quinazoline derivatives presented promising VEGFR-2 inhibition activity. They used a multi-step approach, starting with easily accessible vanillin **222** and 3-bromophenol **223**, which were converted into 3-bromo-4,5-dimethoxyphenylamine **224** and a boronic ester **225**, respectively. Suzuki coupling of these two intermediates led to the formation of intermediate **226**, which was then treated with triphosgene and substituted anilines, generating urea-bearing compounds **227**. Subsequent deprotection and condensation with hydroxylamine generated library **228** (25 examples) (Scheme 31B). Three compounds (**228a**–**c**) displayed VEGFR-2 inhibition, with IC_50_ in the low nanomolar range (4.2–8.7 nM versus the reference compound sorafenib (Figure 1) 2.2 nM). Interestingly, researchers explored the activity of some analogs not bearing the phenol group (with a methoxy group instead), which presented a much higher IC_50_ value, showcasing the relevance of the intramolecular hydrogen bond. Derivatives **228a**–**c** also proved to be efficient antiproliferative agents against different tumor cell lines, with the best results achieved against H1299 for **228a** (IC_50_ of 2.63 μM), MDA-MB-435S for **228b** (IC_50_ of 0.35 μM), and A375 for **228c** (IC_50_ of 2.37 μM) [111]. Zhang’s research group also reported on the synthesis of a library of diarylurea derivates (bearing a quinazolinone moiety) and diaryl thiourea derivates (bearing a pyridine moiety). While the thiourea derivatives did not display relevant VEGFR-2 inhibitory activity, most of the urea derivatives displayed moderate to good VEGFR-2 inhibition. The synthetic approach for these diarylurea derivatives consisted of the cyclization of 2-amino-5-bromobenzoic acid **229** to 7-bromoquinazol-4(3H)-one **230** with formamide, which further reacted with a boronic ester **231** via the Suzuki coupling reaction, yielding library **232** (10 examples; see Scheme 31C). Compound **232a** emerged as the most active, showing VEGFR-2 inhibition with an IC_50_ of 0.77 nM, compared to the reference drug sorafenib (Figure 1), which displayed 0.55 nM under the same conditions. Furthermore, this promising molecule proved to exhibit relevant antiproliferative activity in both *in vitro* and *in vivo* cancer models [112].

**Scheme 32:**
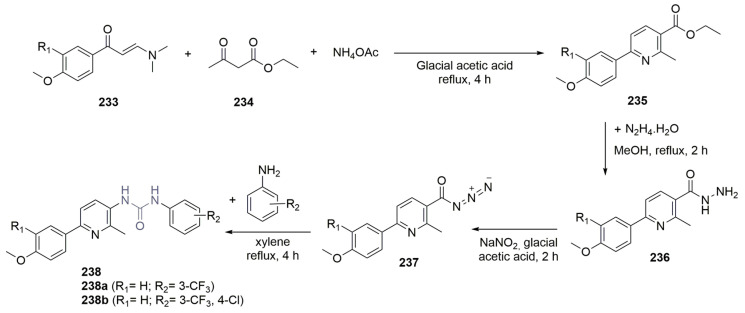
Synthesis of pyridine–urea hybrids with promising antiproliferative activity.

El-Naggar and co-workers explored the molecular hybridization of pyridines and ureas to achieve novel VEGFR-2 inhibitors with promising antiproliferative activity. A multi-step synthetic pathway was developed, comprising a one-pot hetero-cyclocondensation step, involving enaminones **233**, ethyl acetoacetate **234**, and ammonium acetate, promoted by glacial acetic acid under reflux. The resulting esters **235** were then subject to hydrazinolysis to attain hydrazides **236**, which further reacted with sodium nitrite, yielding the corresponding nicotinoyl azide **237**. In the final step, substituted anilines were added under refluxing xylene, yielding library **238** (14 examples) (Scheme 32). Hybrids **238a** and **238b** displayed the most promising profiles, including remarkable antiproliferative activity against the NCI 58 cell line panel (with 43% and 49% growth inhibition percentages, respectively) and VEGFR-2 inhibition in the low micromolar range (IC_50_ values of 5.00 μM and 3.93 μM, respectively). Thus, these inhibition values highlight a potential mechanism of action for these pyridine–urea hybrids [113].

Based on the best scaffolds reported on in the past decade, with strong VEGFR-2 inhibitory activity, some structural features were highlighted. Among the families of potent hybrids (5- and 6-membered heterocyclics, coumarin, isatin, and urea–thiourea), the attached aromatic moieties contributed significantly to enhanced activity. Functional groups such as amides, amines, ethers, and sulfur derivatives were generally present in the hit compounds. Curiously, the urea moiety was present in many of the most potent frameworks as a linker, increasing the bioactivity of the final hybrid compound, likely due to its ability to establish hydrogen bonds with the target site, therefore improving the drug–target interaction. Interestingly, hybrid compounds with the corresponding thiourea framework did not exhibit activity regarding the VEGFR-2 inhibition assay.

## 3. Conclusions

In this work, we highlighted the recent advances in the development of novel VEGFR-2 inhibitors. Numerous studies have reported the discovery of new small-molecule inhibitors with promising antiproliferative activity associated with VEGFR-2 inhibition as a mechanism of action. The molecular hybridization of relevant scaffolds and chemical moieties has proven to be a successful strategy for attaining bioactive molecules. Privileged structures, such as coumarins, oxindoles, and other relevant heterocycles, as well as important functional groups, such as urea, are valuable starting points for structure-based drug design, which can lead to the identification of promising inhibitors.

Despite the progress, ongoing research remains critical to fully exploit the therapeutic potential of VEGFR-2 inhibition. As cancer is an ongoing concern with great socioeconomic impact, it is essential for researchers to focus on optimizing new molecules for clinical applications, improving their safety profiles, and reducing the likelihood of resistance development. The current drug design methodologies are highly focused on the use of fragments of well-known VEGFR-2 inhibitors. Future studies might integrate computational modeling, high-throughput screening, and machine learning or artificial intelligence-assisted drug discovery processes, in order to refine hit-to-lead optimization. Better pharmacokinetic profiles and less off-target side effects might also be promoted by exploring innovative synthetic strategies, namely allosteric modulation, in order to provide more selective, effective, and safer treatment options for cancer patients, ultimately leading to improved clinical outcomes.

## Data Availability

Not applicable.
